# Classification and Evaluation of Octopus‐Inspired Suction Cups for Soft Continuum Robots

**DOI:** 10.1002/advs.202400806

**Published:** 2024-06-14

**Authors:** Stein van Veggel, Michaël Wiertlewski, Eugeni L. Doubrovski, Adrie Kooijman, Ebrahim Shahabi, Barbara Mazzolai, Rob B. N. Scharff

**Affiliations:** ^1^ Department of Sustainable Design Engineering Delft University of Technology Delft 2628 CE The Netherlands; ^2^ Cognitive Robotics Department Delft University of Technology Delft 2628 CD The Netherlands; ^3^ Bioinspired Soft Robotics Laboratory Istituto Italiano di Tecnologia Genoa 16163 Italy; ^4^ Division of Integrative Systems and Design The Hong Kong University of Science and Technology Clear Water Bay Hong Kong China

**Keywords:** biomimetics, octopus sucker, soft robotics, suction cup

## Abstract

The emergence of the field of soft robotics has led to an interest in suction cups as auxiliary structures on soft continuum arms to support the execution of manipulation tasks. This application poses demanding requirements on suction cups with respect to sensorization, adhesion under non‐ideal contact conditions, and integration into fully soft systems. The octopus can serve as an important source of inspiration for addressing these challenges. This review aims to accelerate research in octopus‐inspired suction cups by providing a detailed analysis of the octopus sucker, determining meaningful performance metrics for suction cups on the basis of this analysis, and evaluating the state‐of‐the‐art in suction cups according to these performance metrics. In total, 47 records describing suction cups are found, classified according to the deployed actuation method, and evaluated on performance metrics reflecting the level of sensorization, adhesion, and integration. Despite significant advances in recent years, the octopus sucker outperforms all suction cups on all performance metrics. The realization of high resolution tactile sensing in suction cups and the integration of such sensorized suction cups in soft continuum structures are identified as two major hurdles toward the realization of octopus‐inspired manipulation strategies in soft continuum robot arms.

## Graphical abstract

This review discusses the state‐of‐the‐art in octopus‐inspired suction cups for soft robots. A comprehensive biological analysis of the octopus sucker forms the basis for deriving metrics for evaluating the adhesion performance and sensory innervation of suction cups. 47 research works on suction cups are categorized by actuation technology and evaluated on the defined metrics. Finally, the limitations and opportunities toward narrowing down the gap with the octopus sucker performance are discussed.

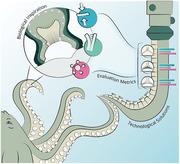

## 1 Introduction

Suction cups have been around for centuries, with the medical use of suction cups being already mentioned in the Ebers papyrus (1550 BC) and Hippocratic corpus,^[^ ^1^ ^]^ and the first patents making use of suction cups dating back to the 1860s.^[^ ^2^, ^3^ ^]^ Nowadays, suction cups are still commonly used for a variety of applications such as the unclogging of drains and the picking and placing of objects for manufacturing and warehouse automation. Throughout the past century, the design of the suction cup has remained mostly the same with only a few innovations since the early days of patents. However, the recent emergence of soft robotics has led to a renewed interest from the academic community in suction cups. In an attempt to mimic the manipulation capabilities of octopus arms, researchers have integrated suction cups on soft continuum robot arms.^[^ ^4^ ^]^ This application poses several demanding requirements for suction cups.

First, in the absence of vision, the octopus heavily relies on information from the (chemo‐)tactile receptors in its suckers to successfully complete tasks.^[^ ^5^ ^]^ The mimicking of such octopus strategies in soft continuum robot arms therefore requires sensorization of suction cups.

Second, the unstructured environments in which these soft continuum robot arms are intended to operate often results in non‐ideal contact between the suction cup and the object of interest. The suction cup may approach the object at an angle, the object may have a challenging geometry or texture, and the preload that can be applied is limited by the softness of the arm. More robust suction cup designs are required to ensure a good sealing performance and consequent adhesion under a variety of conditions. Third, the integration of a large number of suction cups on a soft continuum robot arm poses significant challenges with respect to the size and softness of the suction cups, as well as their power and control system.

A large body of recent research work has focused on addressing these key challenges, with the octopus sucker as a key source of inspiration. However, roboticists often face difficulties in identifying and interpreting relevant findings from biological studies on the octopus sucker. Moreover, existing attempts to mimic the functionality of octopus suckers in suction cups lack a unified framework to evaluate the suction cups' performance. This review aims to accelerate this line of research by providing the reader with a detailed analysis of the octopus sucker as well as an overview of the existing attempts to mimic its functionality in suction cups. Through quantitatively comparing the performance of the octopus sucker to the state‐of‐the‐art suction cups, this work highlights the main performance gaps and provides a perspective on promising directions toward addressing these gaps. The focus of this review is on the sucker of the Octopus vulgaris. Other works that review research on octopus‐inspired robotics exist, but have a substantially different scope. Giordano et al. have analyzed the capabilities of octopus skin and mapped promising technologies for mimicking these capabilities.^[^ ^6^ ^]^ Another review effort is the work by Bagheri et al.^[^ ^7^ ^]^ This broader review work also includes a qualitative overview of actuation mechanisms for suction cups but does not provide an in‐depth analysis of how the challenges of sensing, adhesion, and integration are addressed. In this work, we provide a comprehensive and quantitative analysis of the performance of biological as well as artificial suction cups.

The review work is structured as follows. Section 2 provides an overview of research on the morphology, adhesion mechanisms, tissue properties, tactile sensing mechanisms, and system‐level function of octopus suckers. In Section 3, this overview is used as a basis to define several sets of metrics to evaluate and compare suction cups. After applying a structured search method as described in Section S1 (Supporting Information), a broad range of records that describe one or more suction cup designs was obtained. In Section 4, these suction cup designs are classified first by their actuation technologies. Then, the sets of metrics are used to evaluate the state‐of‐the‐art following a similar structure as used for the octopus sucker biology, covering their architecture and actuation, adhesion strategies, manufacturing processes, and materials, tactile sensing mechanisms and their (potential) integration and control on a system‐level. Section 5 reflects on the results, elaborates on limitations, and identifies promising research directions to advance the capabilities of suction cups for application in soft continuum arms. The conclusions of the review article are presented in Section 6.

## 2 Octopus Suckers

The octopus sucker is a remarkable structure that is used for 1) locomotion, 2) anchoring the body and holding prey, 3) sampling, collecting, and manipulating small objects, 4) chemotactile recognition, 5) behavioral displays, and 6) cleaning manoeuvres.^[^ ^8^ ^]^ The sucker morphology enables reversible adhesion on rough, curved, and deformable surfaces, while also allowing for modulation of the attachment force in response to changing environmental conditions.^[^ ^9^ ^]^ Negative pressures up to 0.268 MPa can be achieved in the octopus sucker in a matter of milliseconds.^[^ ^10^ ^]^ This section focuses on describing the biological mechanisms that underlie the realization of these desirable features. Emphasis has been put on those mechanisms that can serve as an inspiration for the design of suction cups. Specifically, this section looks into the sucker morphology (Section 2.1), adhesion (Section 2.2), tissues (Section 2.3), chemo‐tactile sensing capabilities (Section 2.4), and neural integration (Section 2.5).

### 2.1 Morphology

**Figure 1:**
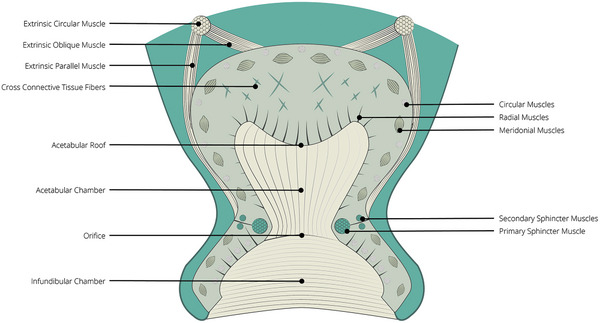
Morphology of the octopus sucker and its terminology.

The octopus sucker consists of two regions, the infundibulum and the acetabulum. The infundibulum is responsible for conforming to the substrate shape and forming a tight seal, while the acetabulum is the chamber that is responsible for creating the pressure difference with respect to the external environment. The two chambers are connected through an orifice, which is shown in **Figure** **1**.^[^ ^11^ ^]^ Octopus suckers are muscular hydrostats with muscle arrays oriented along three dimensions. This way, the muscles both generate the force and provide the support for movement. The muscle arrays are 1) radial muscles that traverse the sucker wall, 2) circular muscles, arranged circumferentially, and 3) meridional muscles, perpendicular to the radial and circular muscles. This is shown schematically in Figure 1. Through precisely localized deformations, the infundibulum shape can closely match the surface contours of the substrate and ensure a watertight seal. The suckers are attached to the arm by a short muscular base with extrinsic muscles. This base enables the octopus to rotate and elongate the sucker as a whole and transmit the attachment force to the arm. This way, attached objects can be easily manipulated after attachment. Similar to the intrinsic sucker musculature, the extrinsic muscles are oriented in three directions, oblique, parallel, and circular.^[^ ^11^ ^]^ These muscles are also shown in Figure 1.

### 2.2 Adhesion

**Figure 2:**
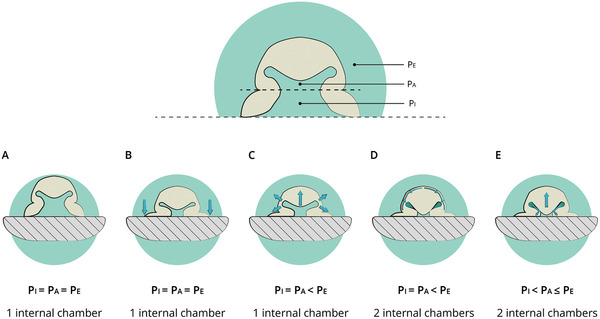
Visual description of the adhesion process of an octopus sucker in five steps. A) Initial contact with the substrate, B) formation of a seal after contracting the infundibular radial muscles, C) pressure reduction through contraction of the acetabular radial muscles, D) interlocking of the protuberance in the orifice through contraction of the meridional muscles, and E) Continued adhesion after relaxing of the radial muscles due to the friction from hairs and ridges, cohesive forces of water and elastic energy in the cross connective tissue fibres.

Octopuses employ their suction cups for grasping and manipulation of objects with varying shapes and sizes, which is achieved through the process of adhesion. The adhesion process consists of the following steps. After achieving contact with the substrate (see **Figure** **2**A), the infundibular radial muscles contract. As contraction in one direction has to be balanced by elongation in the other, this increases the infundibular surface area.^[^ ^10^ ^]^ The infundibulum actively matches the shape of the substrates and forms a seal (see Figure 2B). Then, the acetabular radial muscles contract to thin the acetabular wall and increase the cavity volume. However, the cohesiveness of water resists this expansion, so the pressure is reduced in this process^[^ ^9^ ^]^ (Figure 2C). After that, the meridional muscles bring the acetabular protuberance down and interlock it in the orifice. This creates a water torus (see Figure 2D). The acetabular radial muscles stop contracting but the friction from the hairs and ridges, cohesive forces of water, and elastic energy in the cross‐connective tissue fibres prevent the protuberance from going back to its original position (see Figure 2E). Because the radial muscles are not contracted anymore, the pressure in the water torus increases again while the infundibular pressure remains low. Lastly, contraction of the circular muscles releases the acetabular protuberance from the orifice and detaches the sucker from the substrate.^[^ ^12^ ^]^

**Figure 3:**
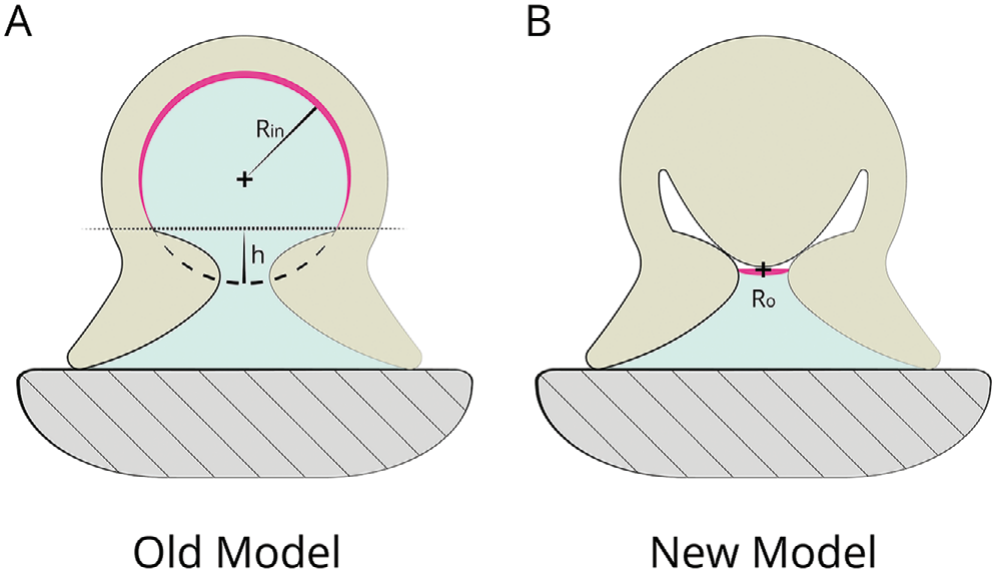
Visual explanation of how the new adhesion model as proposed by Tramacere et al.^[^ ^10^ ^]^ leads to a smaller surface of action.

During the adhesion process, the octopus possesses a peculiar mechanism to save energy during extended periods of suction. Kier et al.^[^ ^9^ ^]^ suggested that the cross‐connective tissue fibers in the acetabular roof, shown in Figure 1, play a role in storing elastic energy. The mechanism is assumed to work as follows. Prior to attachment, the meridional and circular muscles contract to thicken the acetabular roof. This creates a pre‐strain in the cross‐connective tissue fibres. When this force is removed, the stored elastic energy tends to thin the wall in a similar way the radial muscles do. The radial muscles can then be relaxed to save muscular energy.^[^ ^9^ ^]^ After relaxing the radial muscles, the protuberance remains interlocked due to the cohesive forces of water and the friction force produced by the surface texture of the acetabular protuberance. These forces are counterbalanced by the elastic force in the cross‐connective tissue. An additional advantage is that the surface of action is lowered (see **Figure** **3**A for the old model, and Figure 3B for the new one), thereby decreasing the force needed to achieve the same negative pressure.^[^ ^10^ ^]^

### 2.3 Tissue Properties

**Figure 4:**
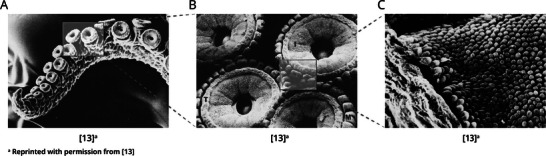
Electron microscope images of the sucker tissue. In the middle image, the radial grooves are clearly visible, while the right image displays the microdenticles on the infundibular surface.^[^ ^13^ ^]^

In addition to the adhesion process, investigation of the sucker's surface‐ and mechanical properties are equally important for designing suction cups. Graziadei^[^ ^13^ ^]^ looked at the infundibulum surface using electron microscopy (EM) and light techniques and discovered that its tissue consists of two regions. The outer edge consists of smooth tissue and is assumed to be responsible for forming the watertight seal. The inside of the surface consists of radially lined grooves (see **Figure** **4**B). Through these radial grooves, the sub‐ambient pressure is divided over a greater area. As the attachment force is the product of the pressure in the sucker cavity and the area of attachment, this principle increases the sucker force significantly. Moreover, the grooves increase friction between the sucker and the substrate, which makes it resistant to both tensile and shear forces. When zooming in further, microdenticles are found on the infundibular surface (see Figure 4C), which further contribute to this purpose. Without these microproperties, the seal would only be formed at the orifice and no force would be available to hold the infundibulum against the substrate.^[^ ^11^ ^]^ Tramacere et al.^[^ ^14^ ^]^ investigated the mechanical properties of the sucker in several indentation experiments. They proposed that the acetabulum is elastic and hard, which makes it contribute to efficient on/off attachment, storage of elastic energy, and prevention of collapse of the suction chamber during negative pressure generation. The infundibulum, however, is soft, visco‐elastic, and compliant. This makes sure it can conform to several kinds of shapes. It has even been observed that the epithelium of the infundibulum surface secretes mucus, which further contributes to the adhesion and watertight seal.^[^ ^11^ ^]^

### 2.4 (Chemo‐)Tactile Sensing

**Figure 5:**
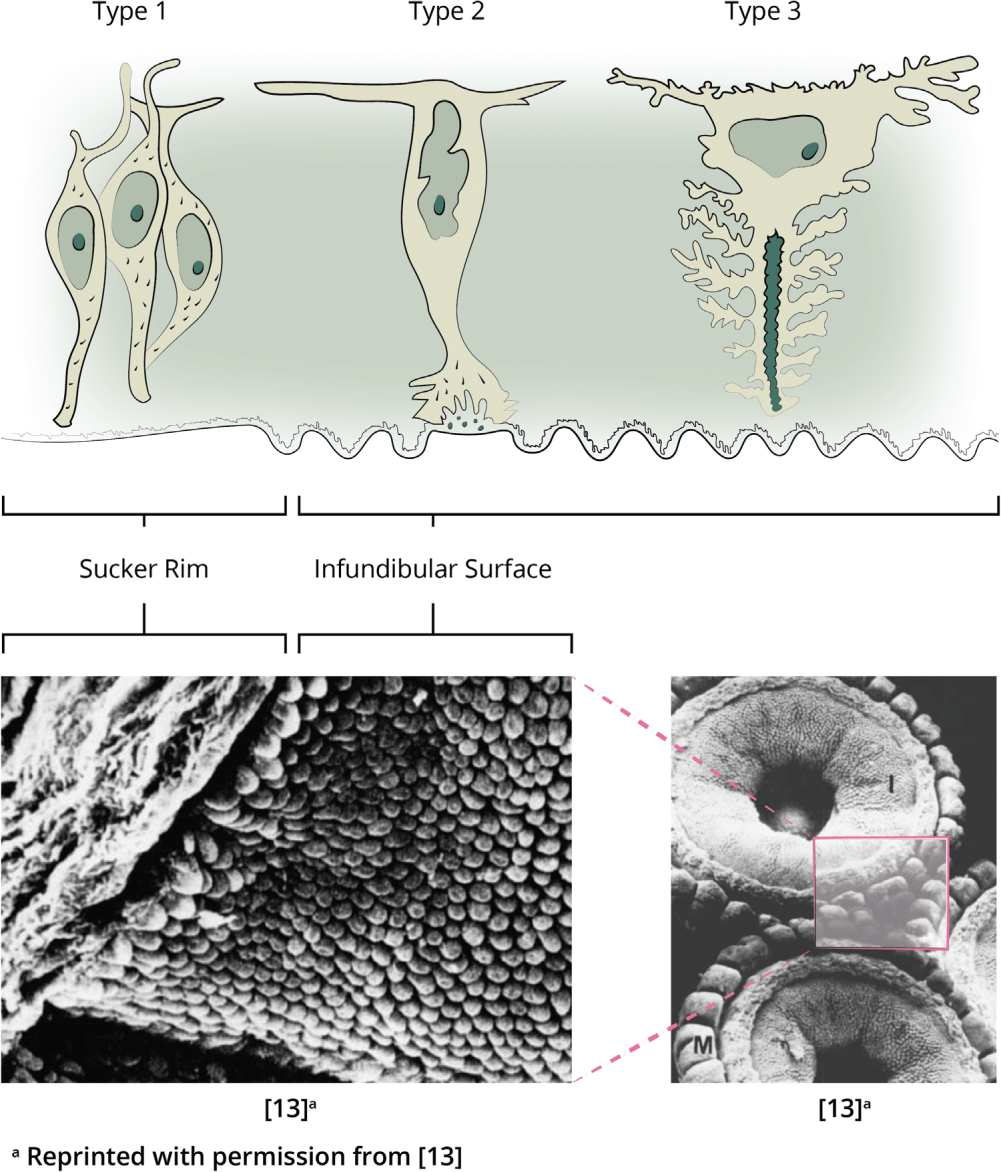
Three different types of sensors located on the octopus sucker rim and infundibular surface, as discovered by Graziadei.^[^ ^13^ ^]^

Graziadei and Gagne^[^ ^13^ ^]^ studied the anatomy and distribution of sensory receptors in octopus suckers using light and EM studies. They found that a single sucker of 3 mm in diameter contains tens of thousands of receptors, mostly located on the infundibular surface. They found three categories of sensory receptors in the sucker epithelium, as shown in **Figure** **5**: 1) Long, ciliated cells, assumed to be chemoreceptors and mostly located at the sucker edge, 2) fusiform and terminating in a pore, assumed to work as both chemo‐ and mechanoreceptor for sensing contact, and acting to sense pressure and normal deformation, and 3) rounded cells with dendrites, assumed to be mechanoreceptors and likely responsible for sensing lateral sucker deformation and checking whether or not a seal has been formed. While most receptors are evenly distributed along the infundibular surface, the acetabular receptors are mostly concentrated at the protuberance.^[^ ^15^ ^]^ As this part of the sucker plays a role in sealing the chamber, it is likely that these receptors may have a function of sensing the internal pressure changes.

### 2.5 Neural Integration and Control

**Figure 6:**
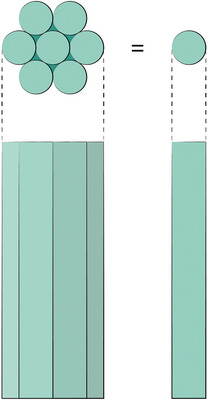
Two cylinders that appear identical to octopuses when sampling the surface with their suckers according to the experiments of Wells.^[^ ^17^ ^]^ The reason is that they have similar local surface curvatures.

The sensing ability of the suckers can be approached more holistically by examining how the information is used on a system level. Two primary abilities can be identified. First, the sensed information is integrated to form a representation of external objects and the environment, by the suckers forming a topologically ordered spatial array on the arm. Touching a surface with multiple suckers enables the octopus to form a representation of shape, curvature, and texture. Röckner et al.^[^ ^15^ ^]^ observed that this function is facilitated by quick and loose attachment and detachment patterns that they identified as orientation contraction attachment. It has even been observed that octopuses sometimes conform to surface shapes with only the rim in contact, without forming a seal, indicating predominantly sensory and exploratory purposes. Second, processing the sensed information facilitates the monitoring of precise arm movements and sucker activation patterns.^[^ ^16^ ^]^ Wells^[^ ^17^ ^]^ conducted several behavioral experiments with octopuses that show the role of suckers in estimating arm position. He showed that objects with the same ratio of flat surface to corner are considered identical, as explained in **Figure** **6**. In other words, rough objects with large radii produce similar sensory inputs as smooth objects with small radii. This concludes that the octopus may not be able to recognize the global position and shape of their suckers, and can therefore not be using any input from proprioceptors in their arms. Object diameters are thereby only judged from local surface curvature, measured by the distortion degree of the sucker sampling the surface.

In extension of the behavioral work of Wells,^[^ ^17^ ^]^ Grasso^[^ ^16^ ^]^ has performed substantial research on the octopus suckers and arms in the scope of neural connections and their representation in the octopus brain. He found that octopuses lack the neural anatomy to form a somatotopic map of their arms in their central brain. In other words, the information coming from the proprioceptors in their arm and suckers can not be translated to communicate the actual arm position, which is in line with Wells's behavioral experiments. This is mainly due to the abundance of parameters. In contrast to vertebrates, cephalopods have completely soft bodies. Hence, they can find themselves to be in many more different states. Grasso mentions that even if the actions of the octopus arm are limited to 1) suckers that can only be attached or free, and 2) sections of the arm that link each pair of suckers to one pitch, one yaw and one roll, such an arm can be in 1.2*10^24^ states.^[^ ^16^ ^]^ It is presumed that the octopus has overcome this hurdle by having a separate “brain” for every arm, which greatly reduces the number of control parameters as controlled by the central brain. This is supported by the fact that an amputated octopus arm is still able to exhibit similar reflexes and movement patterns and that three‐fifths of the totality of neurons are found in the arms.^[^ ^18^ ^]^ Even decerebrated octopuses can still perform tactile discrimination and manipulation tasks. Hence, a somatotopic map might be formed within the arms itself.^[^ ^16^ ^]^ Young's research showed that stimulation of certain brain parts could only generate complex movement patterns such as swimming and walking and that generating movements in only one arm was not possible.^[^ ^18^, ^19^ ^]^ This argues for the fact that details of the movement patterns are located in the arm nervous system, whereas the central brain is only in control of selecting, initiating, and terminating a certain behavior or action.^[^ ^18^, ^20^ ^]^

**Figure 7:**
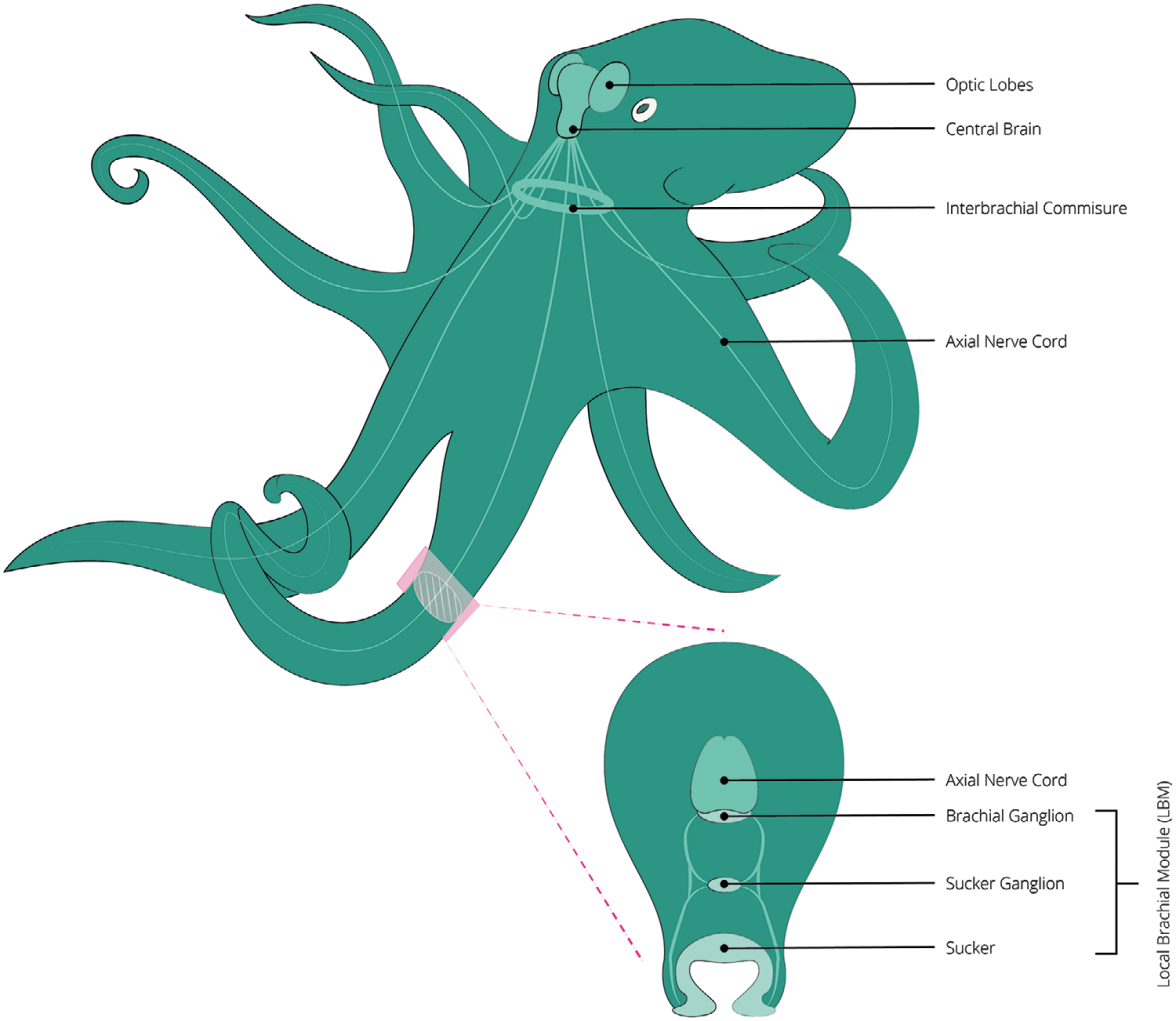
Hierarchical structure of the neural anatomy in the octopus, as described by Grasso et al.^[^ ^16^ ^]^

Considering the suckers specifically, both local (e.g., activating neighboring suckers to concentrate forces) and distant (e.g., alternating activation to “walk” along a surface) activation patterns were observed with precise timing, indicating rich forms of information sharing along the arm. Grasso argues that this behavioral observation indicates pro‐active rather than reflexive control of suckers.^[^ ^21^ ^]^ Anatomically, this is backed up by a hierarchical organization of neurons, with feedback loops forming on multiple levels. On the first and second levels, every sucker is part of its respective local brachial module, which consists of the sucker neurons and sensory receptors, the sucker ganglion, and the brachial ganglion, as shown in **Figure** **7**. By interconnection of the brachial ganglions of every sucker, a chain is formed along the arm. On the third level, one axial nerve cord in each arm could be considered the “arm brain”, which functions as a high‐level neural center within each arm, to integrate information from both the neurons in the arm and the central brain. Finally, the interbrachial commissure interconnects all axial nerve cords by forming a ring of fibres along them.^[^ ^16^ ^]^

To conclude this section, the presumed absence of proprioception and somatotopic representation in the central brain argues the importance of the sucker's sensing abilities. As many arm movements occur outside the octopus's field of vision, the sensing in their suckers is not only needed for exploration of their environment but also for enabling precise control over their arm movements and sucker's activation patterns.

## 3 Performance Metrics

Based on insights gained from studying octopus biology, a potential avenue for future research is the development of soft robotic arms with integrated suction cups and hierarchical control architectures. Such systems could enable the manipulation of a wide range of objects in unstructured and confined environments. The performance metrics were defined with this application in mind.

Four categories of metrics were used to evaluate the performance of the suction cups. The first category consists of nine general performance metrics relating to the main architecture. First, The suction cup diameter (1.1) is reported. Second, the preload (1.2), which was defined as the compressive force required to obtain a seal when pushing the suction cup onto an object. For the integration of suction cups into a soft arm, which may not always be able to deliver this preload, high preloads are beneficial. Third, the maximum attachment force (1.2), which should be as high as possible to be able to grasp heavy objects and resist external forces during attachment. It should be noted that this only includes the attachment force normal to the substrate. The fourth, force by area (1.4) is included as a normalized comparison of the force metrics across suction cups. Next, the response time (1.5), which is defined as the time between the actuation onset and obtaining the attachment force. This should be as low as possible for efficient system‐level manipulation. Metrics 1.6 and 1.7 are the ability to function in dry and wet environments, respectively. Ideally, both are possible here. Performance metric 1.8 describes whether or not detaching the cup can be actively controlled, rather than only enabling detachment by applying a tensile pull‐off or a peeling force. This controllable detachment would again be ideal for integration in a soft system, which may not always be able to deliver these kinds of forces. Also, this precise controllability opens up more possibilities in handling fragile objects, for which high pull‐off forces could bring damage. Finally, for extended periods of suction, it is beneficial if the suction cup, equivalent to the octopus sucker, does not actively consume energy while attached (1.9).

The second category consists of metrics relates to surface adhesion. First, the ability of the suction cup to adhere on curved (2.1), rough (2.2), and soft (2.3) surfaces was evaluated. The suction cup's resistance to shear force (2.4) was included in this evaluation as well. To investigate the adhesion performance of the suction cups in more detail, an analysis of commonly applied adhesive strategies was conducted. Five mechanisms employed by the octopus to enhance adhesion were identified as a framework for guiding the investigation. First, a separation or gradient in mechanical properties between the sucker surface and the suction chamber (2.5). For example, the division between a softer infundibulum and a more rigid acetabulum, present in the octopus sucker, is able to overcome the trade‐off between obtaining high‐pressure differences without collapsing and compliance to a broad range of shapes. Second, application of surface microstructures in the membrane surface (2.6), for which the octopus sucker has microdenticles in the infundibulum. Third, surface geometry (2.7), similar to the radial grooves and slits present in the octopus sucker. Additional mechanisms for adhesion present in the state‐of‐the‐art was included as well.

The third category looks further into the sensing and control abilities of the suction cups. The set of metrics consists of integration of tactile sensing (3.1), use of the sensed information for closed‐loop control (3.2), and demonstration of integration in a larger robotic (3.3). The first metric was subdivided into three sub‐metrics. These are the number of information channels (3.1.1), the softness of the suction cup and sensing module (3.1.2), and the compactness of the suction cup and sensing module (3.1.3). The number of information channels is there to give a quantitative value to the spatial sensor resolution. The latter two were included to describe the degree of actual “integration” of the sensing module in the soft architecture. As explained by Wang et al.,^[^ ^22^ ^]^ the fabrication method and materials of soft sensing methods should allow the sensor to be a part of the robot body. Ideally, the sensors should originate from the soft robot architecture or should be designed in such a way it does not inhibit its size, adhesion, and mechanical performance.

Finally, in order to further evaluate the integration, or potential integration of the suction cup in a soft robotic arm, the records that include any form of tactile sensing and/or closed‐loop control in their design, were evaluated on six metrics that further cover the suitability of the design to be multiplied, and integrated onto a soft robot arm inspired by the octopus arm. The first three metrics are the sub‐metrics 3.1.1 – 3.1.3 that were described above. However, rather than a binary score, the designs were scored between one and six to provide a more nuanced score. A total of six metrics were used: (4.1) The number of information channels, for which it was assumed that a higher number of channels indicates a better spatial sensing resolution, (4.2) the size of the suction cup and the sensing module together, where larger sizes limit the potential for further integration, (4.3) the softness of the suction cup and sensing module, as methods that employ more rigid materials would limit the deformation properties of both the suction cup and soft arm, (4.4) the simplicity of the overall design, as increasing the number of suction cups on the robotic arm should be as easy as possible, (4.5) endurance of the suction cup and sensing module in harsh environments, as it is necessary to be able to operate in a wide range of operating contexts, and (4.6) directness of the measurement in relation to the shape of the contact surface between the suction cup and substrate, for which it was assumed that a more direct measurement can provide more accurate information about both the shape of the suction cup and the substrate.

## 4 Suction Cups

**Figure 8:**
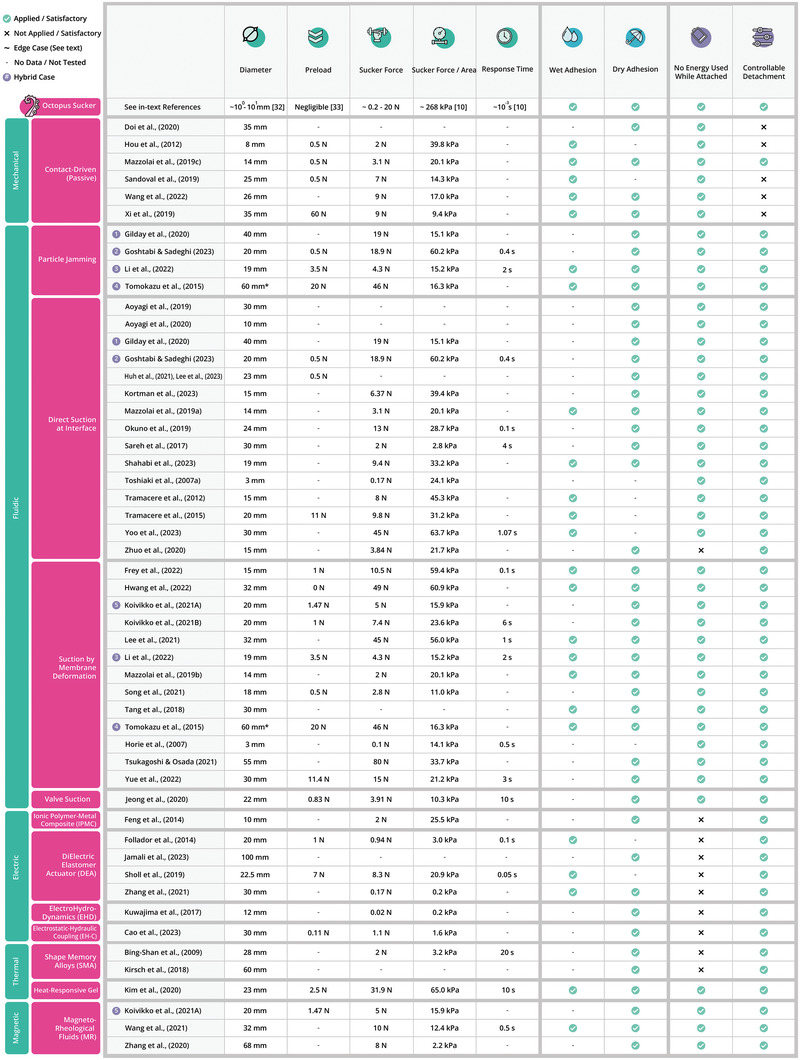
Rating of suction cup designs on general performance metrics relating to the main architecture, classified by their actuation principles. The top row displays a comparison to these metrics in the octopus sucker.^[^ ^10^, ^32^, ^33^ ^]^

In total, 47 records were reviewed for the evaluation metrics described in Section 3. The years of publication of the records ranged from 2007 to 2023. The results are summarized in **Figures** **8**, 10, and 12. The vertical axis of each table classifies the records according to their actuation method. For comparative purposes, the first column provides the performance of the actual octopus sucker.

**Figure 9:**
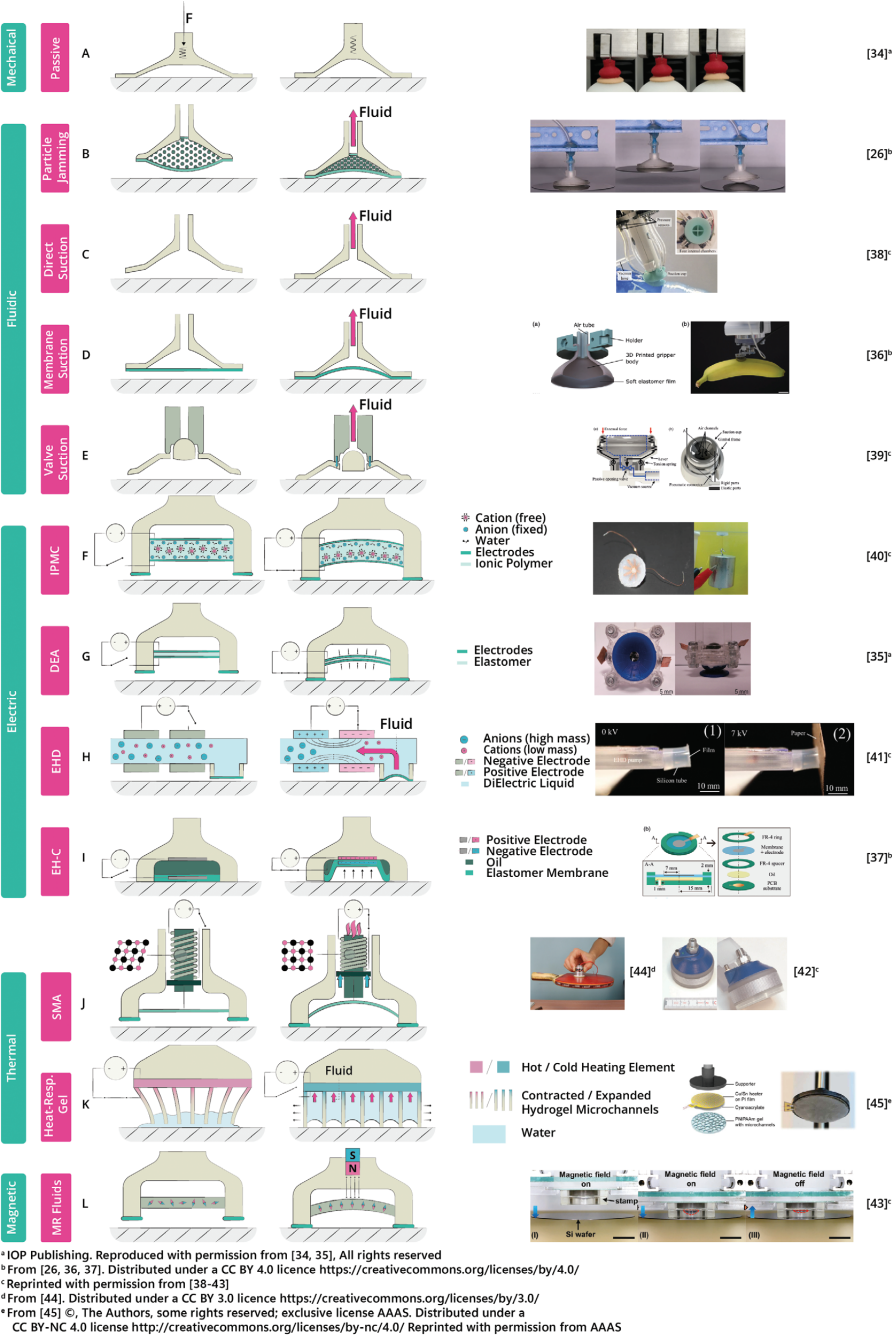
Visual explanation of the working principles of the actuation methods, elaborated with examples from records.^[^ ^26^, ^34^, ^35^, ^36^, ^37^, ^38^, ^39^, ^40^, ^41^, ^42^, ^43^, ^44^, ^45^ ^]^

**Figure 10:**
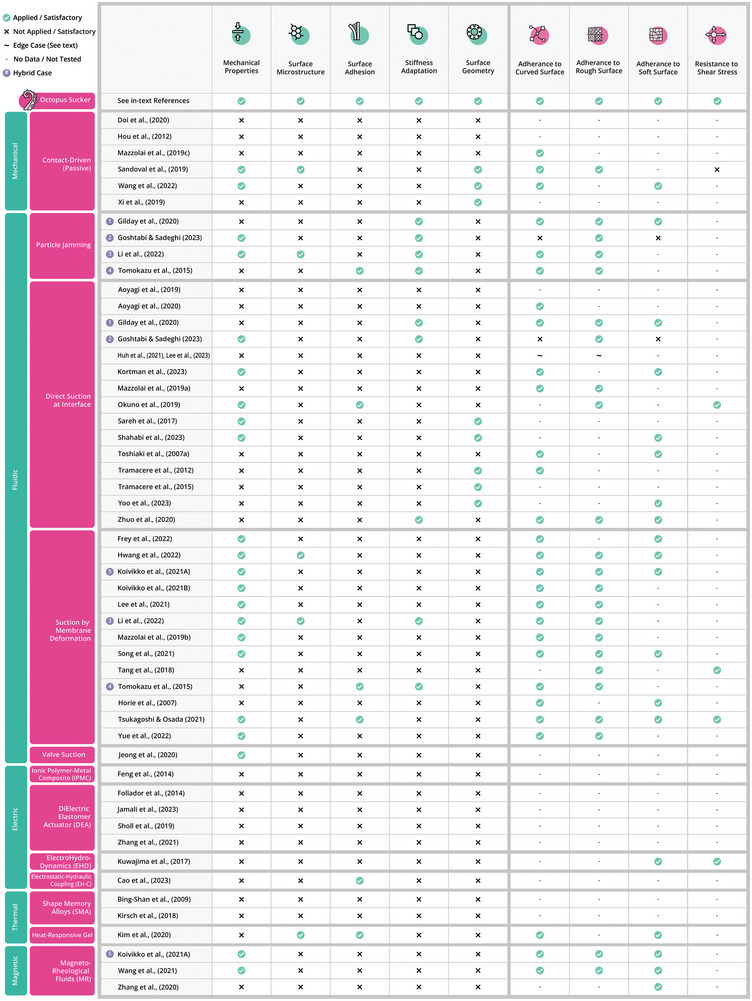
Evaluation of suction cup designs on surface adhesion metrics and investigation of strategies for improved adhesion performance, classified by their actuation technologies. The top row displays the outcomes of these metrics in the octopus sucker.

**Figure 11:**
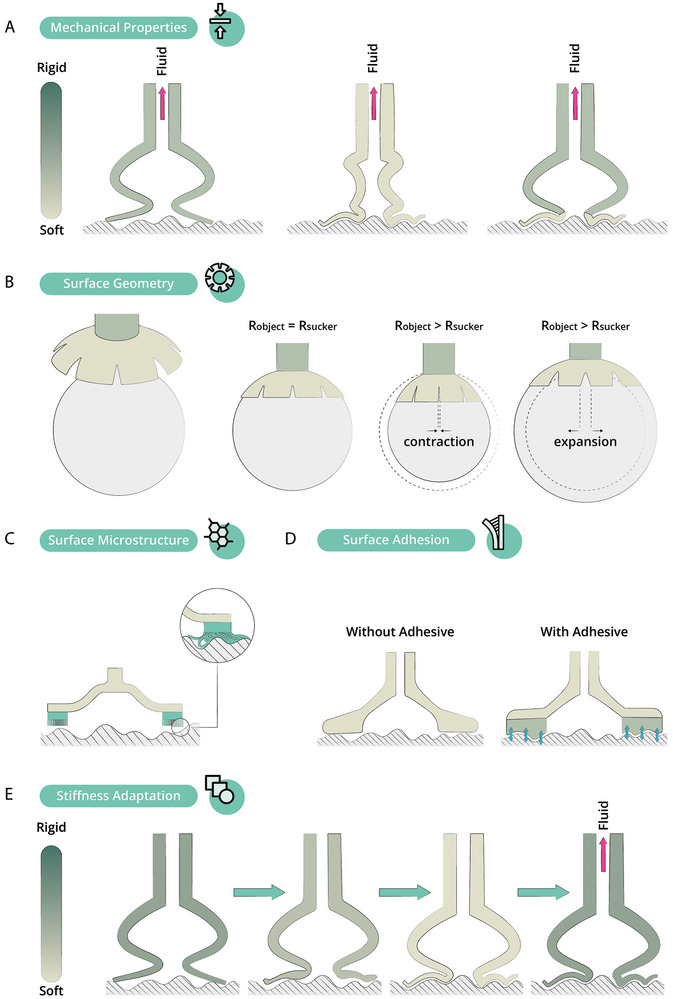
Visual explanation of principles applied for improved adhesion on several types of substrates. A) Mechanical properties, B) surface geometry, C) surface microstructure, D) surface adhesion, and E) stiffness adaptation.

**Figure 12:**
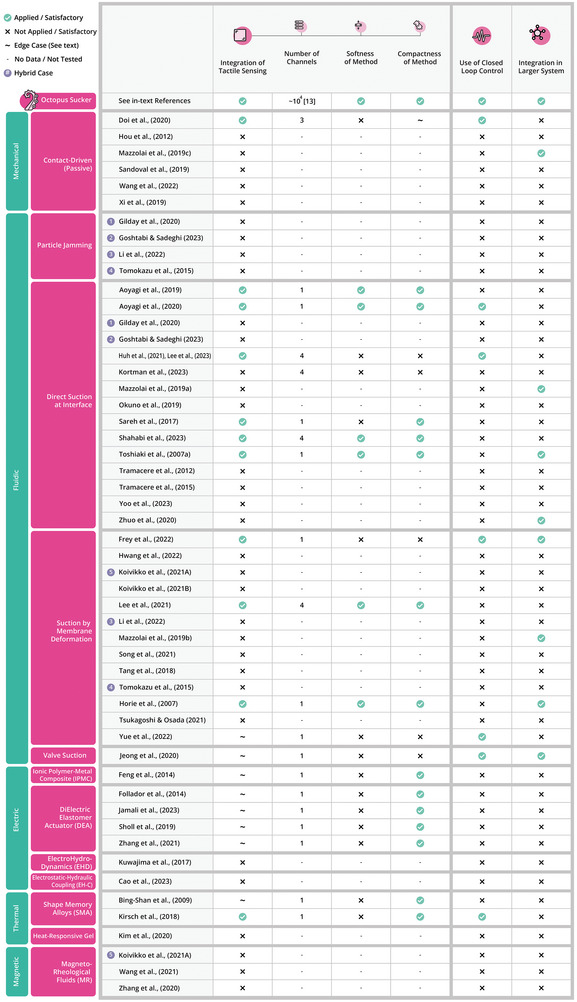
Evaluation of suction cup designs on metrics related to tactile sensing, control & system, integration, classified by their actuation technologies. The top row displays the outcomes of these metrics in the octopus sucker.^[^ ^13^ ^]^

To categorize the different actuation methods, inspiration has been drawn from studies that present similar frameworks to organize actuation methods for soft robot grippers. For instance, Shintake et al.^[^ ^23^ ^]^ and El‐Atab et al.,^[^ ^24^ ^]^ have each presented frameworks to sort actuation methods based on their designs and functional characteristics. The framework here has been adapted to fit suction cups. The categories used are mechanical, fluidic, electric, thermal, and magnetic. Five records are put in twice as they rely on hybrid actuation.^[^ ^25^, ^26^, ^27^, ^28^, ^29^ ^]^ Two records described multiple suction cup designs that could all be placed in different actuation categories.^[^ ^30^, ^31^ ^]^ The details of the search strategy, the classification procedure, and the evaluation method can be found in Sections S1, S2, and S3 (Supporting Information). Additional details about the records are to be found in the raw data table in Section S5 (Supporting Information). The rest of this section describes the state‐of‐the‐art in suction cups. The subdivision is structured in a similar way as the octopus sucker biology in Section 2. First, the architecture and actuation technologies of the suction cups are described, including their advantages and disadvantages. Then, the strategies for adhesion, materials and manufacturing, tactile sensing, and system integration & control are described.

### 4.1 Architecture and Actuation

**Figure** **9** illustrates the working principles of the actuation technologies deployed in suction cups and highlights an example for each of them. This subsection discusses each actuation technology in more detail.

#### 4.1.1 Passive Methods

In suction cups that rely on actuation by an external motor, shape deformations are driven by contact with the substrate. First, the suction cup must be pressed onto the substrate with a certain preload, which results in deformation and obtaining the seal with the substrate. This deformation initially leads to a decrease of the volume in the suction chamber. The elastic restoring force or active retraction of the center then causes a volume increase, and hence a pressure decrease in the chamber (see Figure 9A). Shintake et al.^[^ ^23^ ^]^ mention the high mechanical robustness and maturity of the technology as the main advantages. In addition, the size and weight of the suction cup architecture are independent of the motor, which offers a broad range of motor options for achieving desired performance specifications. Choosing high torque motors can thereby provide high suction cup forces. However, when considering the integration in soft robot arms, this advantage becomes obsolete. These high forces can often not be delivered from soft manipulators. Therefore, the need for a preload makes this technology sub‐optimal for these types of applications. Another drawback, as shown in Figure 8, is that passive suction cups is the only actuation type that don't allow controllable detachment. Instead, a pull‐off or peeling force is required, which brings additional problems when considering fragile objects. Passive suction is applied by six of the records.

#### 4.1.2 Particle Jamming

Whereas passive methods rely on contact to drive deformation and pressure change, fluidic actuation methods use a pump to obtain a vacuum. The first type of fluidic method, worth discussing in a separate section, is particle jamming. This method relies on changes in stiffness. An increase of stiffness is obtained by depressurizing a loose granule‐filled bag. The compressive forces between the particles constrain their movements, making the bag behave as a rigid object (see Figure 9B).^[^ ^23^ ^]^ It should be noted that grippers that solely rely on particle jamming are no longer considered suction cups. Hence, the jamming suction cups included in this overview all deploy a hybrid method, where another actuation principle is responsible for obtaining the actual vacuum between the suction cup and substrate. Advantages of particle jamming include the ability to attach to a large variety of surfaces and fast response times.^[^ ^23^ ^]^ Moreover, traditional suction cup designs typically have a trade‐off between either high forces or good shape adaptability. High forces and rigid holding require good structural integrity with relatively stiff cup materials, to ensure the internal cup shape is preserved. However, cups made from stiffer materials are less able to adapt to non‐smooth and uneven surfaces. The jamming suction cup circumvents this trade‐off by switching between a soft, malleable shape, and a stiff state which holds the shape of the cup when jamming is activated.^[^ ^25^ ^]^ A disadvantage of this type of suction cup is the complicated fabrication.^[^ ^23^, ^46^ ^]^ As a consequence of the manufacturing challenges, particle jamming suction cups are relatively large as can be seen in the diameter column in Figure 8. Four of the records used particle jamming as an (additional) actuation principle. Gilday et al.^[^ ^25^ ^]^ and Goshtasbi & Sadeghi^[^ ^29^ ^]^ combined jamming with direct suction while Li et al.^[^ ^26^ ^]^ and Tomokazu et al.^[^ ^27^ ^]^ combined it with membrane‐based suction.

#### 4.1.3 Other Fluidic Methods

There are three other fluidic actuation methods that share similarities to the extent that they can be described together in this subsection. The first type is the open suction cup where the fluidic medium directly interacts with the substrate (see Figure 9C). Next, there is the suction cup with a membrane in between the fluidic medium and the substrate (see Figure 9D). Here, the pump actuates retraction of the membrane, which in its turn generates a volume increase of the suction chamber. Third, there is the suction cup that uses a valve in between the fluid and the interface (see Figure 9E). The advantages of these methods are quick response times and high forces.^[^ ^23^, ^46^ ^]^ Drawbacks include the need for pumps and pneumatic channels, which increase the total size of the robot manipulator. Other drawbacks are difficult controllability emerging from nonlinear rubber properties^[^ ^23^ ^]^ and difficulty in miniaturization.^[^ ^46^ ^]^ From the three fluidic methods described above, the open suction cup typically shows stronger adhesion than a membrane‐based one,^[^ ^30^ ^]^ as the pressure difference that can be generated by membrane suction cups is limited by the maximum membrane actuation strain. However, several of the membrane suction cups are still capable of achieving high forces. In fact, Figure 8 shows that the highest force of 80 N was achieved by a membrane suction cup.^[^ ^47^ ^]^ The highest reported forces in the table are achieved by the membrane suction cups by Yoo et al.,^[^ ^48^ ^]^ Lee et al.,^[^ ^49^ ^]^ and Hwang et al.^[^ ^50^ ^]^ These generated forces of up to 49 N, and a high force per area of up to 63.7 kPa. It is interesting to note that the latter two designs of Lee et al. and Hwang et al., together with one other record, rely on a pressure increase, rather than a decrease. They all have created strategic geometries where the deflation of certain parts create a volume increase in the suction chamber. An advantage of a membrane‐based approach is the isolation of the fluidic channels from the environment, protecting it from water and dirt.^[^ ^30^ ^]^ Figure 8 indeed shows that most of the membrane suction cups have been tested in wet environments, whereas suction cups using direct suction are often not tested in wet environments. An exception is the design by Tramacere et al.,^[^ ^14^ ^]^ who reported using water as a medium throughout the entire fluidic circuit. Another exception is the work of Mazzolai et al.^[^ ^30^ ^]^ who filled the fluidic channel with the environmental medium (either water, oil, or air) before testing.

#### 4.1.4 Ionic Polymer Metal Composites

Ionic Polymer Metal Composites (IPMCs) consist of an electrolyte‐swollen polymer membrane sandwiched between two electrodes. When a voltage is applied, the cations move to the cathode, and vice‐versa. This leads to differential swelling and causes a bending deformation (see Figure 9F).^[^ ^23^ ^]^ This technology requires relatively low voltages and shows response times in the range of 1–10 s.^[^ ^23^ ^]^ The self‐sensing properties of the material could theoretically be used for strain sensing. In the work of Feng et al.,^[^ ^40^ ^]^ IPMC is used for actuation. The structure bends when a voltage of 3–5 V is applied, and forms a negative pressure by generating an increase of the volume between the substrate and suction cup.

#### 4.1.5 Dielectric Elastomer Actuators

Dielectric Elastomer Actuators (DEAs) are composed of a thin elastomer membrane sandwiched between two electrodes. Upon applying a voltage to the electrodes, the electrostatic attraction between them squeezes the membrane, which results in a shape change.^[^ ^23^, ^24^, ^46^ ^]^ This shape change can be used to increase the volume of the suction chamber and consequently decrease the pressure in the chamber (see Figure 9G). Advantages of DEAs include the fast response time,^[^ ^23^, ^46^ ^]^ (100 ms in Follador et al.^[^ ^35^ ^]^ and 50 ms in Sholl et al.^[^ ^51^ ^]^), low use of energy,^[^ ^23^, ^46^ ^]^ light weight^[^ ^46^ ^]^ and the possibility for self‐sensing of strain by measuring the material resistance.^[^ ^23^, ^24^ ^]^ However, the latter has not been applied in any of the suction cups. Drawbacks of DEAs include the complicated fabrication, hysteresis in the actuation response and low output forces,^[^ ^23^, ^46^ ^]^ which is visible in Figure 8 as well. There is also a safety issue, as there exists a risk of electric discharge outside the actuator.^[^ ^23^, ^24^ ^]^ Additionally, the need for a rigid backbone normally limits integration in a system that is designed to be entirely soft.^[^ ^7^ ^]^ However, this drawback has recently been overcome by Jamali et al.^[^ ^52^ ^]^ by utilizing a flexible silicone ring as a backbone. Four records^[^ ^35^, ^51^, ^52^, ^53^ ^]^ apply DEAs for actuating the suction cup. Follador et al.,^[^ ^35^ ^]^ Jamali et al.,^[^ ^52^ ^]^ and Zhang et al.^[^ ^53^ ^]^ used the material in the cup membrane. A voltage deforms the membrane and increases the volume in the suction cup chamber, which results in a pressure decrease. Sholl et al.^[^ ^51^ ^]^ used the DEA in a different way. Instead of using the material to actuate membrane deformation in order to achieve volume change, the material was coiled inside the flexible walls of a vessel that functions as the suction chamber. Hereby, a higher change in volume and hence a higher output force could be achieved.

#### 4.1.6 ElectroHydroDynamics

The ElectroHydroDynamics (EHD) phenomenon works with obtaining a fluid flow by applying a high‐intensity electric field to a dielectric fluid. The flow emerges because there is a difference in density between the negative and positive particles. This flow results in a pressure decrease (see Figure 9H). Kuwajima et al.^[^ ^41^ ^]^ made use of this phenomenon for creating a vacuum in their suction cup. Using only a 3D printer and cutting plotter, the structure was relatively simple to manufacture. However, a high voltage (6 kV) is required to reach a negative pressure of only 0.6 kPa, obtaining a force of 0.02 N, which is by far the lowest reported in Figure 8.

#### 4.1.7 Electrostatic‐Hydraulic Coupling

Cao et al.,^[^ ^37^ ^]^ utilized an electrostatic‐hydraulic actuation mechanism to create a negative pressure cavity, resulting in an adhesive force of 1.1 N. The mechanism relies on an electrostatic attraction force between two electrodes. The first being the copper plate mounted on the Printed Circuit Board (PCB) that forms the backplate of the suction pad, and the second being the flexible electrode on the membrane made with painted carbon black powder particles. When a voltage is being applied, the attraction force between the membrane and the backplate pushes the dielectric liquid out of the central region to the sides, resulting in a seal and a cavity with negative pressure between the substrate and the suction pad (see Figure 9I). Advantages listed by the authors include a low preload of only 0.11 N and a higher adhesive stress‐to‐power ratio than other smart‐material‐based techniques. The design is also more compact than many other techniques. Their architecture enabled them to obtain a height of only 2 mm in their suction cup of 30 mm in diameter. This gives it a high ratio of force to unit volume. The limitations mentioned are the need for a stiff PCB as a backplate, which makes it difficult to adhere to curved and deformable surfaces. Moreover, even though the ratio of adhesive force to power is high, the actuator requires an actuation voltage of 4 kV.

#### 4.1.8 Shape Memory Alloys

Shape Memory Alloys (SMAs) show a shape memory effect due to the crystallographic change of the alloy between two phases. Heating of the SMA above the transition temperature results in a higher modulus and a recovery of the shape to its initial state. Although this material is mostly used for stiffness modulation, it can also be used to generate a change in shape (see Figure 9J).^[^ ^23^, ^24^, ^46^ ^]^ One of the advantages is that the actuation elements are relatively small.^[^ ^46^ ^]^ Another advantage is the potential self‐sensing property. Measuring the SMA's resistance can be used to deduct the material strain. Drawbacks include slow response times (20 s for Bing‐Shan et al.^[^ ^44^ ^]^),^[^ ^23^, ^24^, ^46^ ^]^ poor fatigue characteristics,^[^ ^46^ ^]^ hysteresis in actuation, difficult shape recovery and high temperatures.^[^ ^23^, ^24^ ^]^ In Figure 8, it is shown that two of the records^[^ ^42^, ^44^ ^]^ use SMAs for actuation. It is interesting to note that Kirsch et al.^[^ ^42^ ^]^ made use of the self‐sensing property of SMAs. This is further discussed further in Sections 4.4 and 4.5.

#### 4.1.9 Thermo‐Responsive Gels

One record deploys a thermo‐responsive gel to actuate a suction cup. Kim et al.^[^ ^45^ ^]^ designed a soft manipulator consisting of a microchanneled hydrogel layer that is actuated by an electric heater on top. Prior to attachment, the hydrogel layer is heated through Joule heating. This causes the hydrogel channels to shrink and drives the water to flow out. Upon switching off the heater, the channels start to expand again, which creates a negative pressure and pulls the water back in. This fluid flow inwards creates an adhesion force between the manipulator and the substrate (see Figure 9K). An advantage of this technique is that the architecture of the hydrogel layer divides the vacuum over a greater number of channels, rather than only having one suction chamber where detachment occurs when the seal breaks. This works similarly to the channel network of grooves and ridges in the octopus sucker, as discussed in Section 2.3. Moreover, using water as a medium between the manipulator and the substrate creates a thin liquid film which improves the seal. The technique requires driving voltages in the range of 2 ‐ 5 kV. As the design by Kim et al.^[^ ^45^ ^]^ applies voltage prior to attachment and turns off the circuit to obtain an adhesion force, the manipulator does not consume energy during attachment. A drawback is that obtaining the force relies on the speed of the cooling process, which can be slow. The authors report that it takes 10 s to achieve proper attachment. Zhuo et al.^[^ ^54^ ^]^ also use thermo‐responsive hydrogels for the actuation of their suction cup. However, here the principle is not used to create the vacuum, but to adapt the stiffness of the suction cup body to be able to adapt to different variations of substrate roughness. This is elaborated on further in Section 4.2.

#### 4.1.10 MagnetoRheological Fluids

Magnetorheological (MR) fluids incorporated into an elastomeric material form chains of ferromagnetic particles along the magnetic flux lines when imposed by a magnetic field, resulting in a shape change (see Figure 9L).^[^ ^23^, ^24^ ^]^ For suction cups using MR particles, the application of a magnetic field causes the deformation of a membrane, which creates an increase of suction chamber volume and a decrease in pressure. Advantages include fast response times and high force generation.^[^ ^23^, ^24^ ^]^ Drawbacks include heat generation, a fabrication process consisting of many steps, and high power consumption.^[^ ^23^, ^24^ ^]^ Additionally, external magnetic coils are usually bulky and thus take up a significant amount of space.^[^ ^24^ ^]^ Some applications might now allow for the generation of a magnetic field near the suction cup. Figure 8 shows that three of the records^[^ ^28^, ^43^, ^55^ ^]^ make use of this principle. Relatively high forces in the range of 5 ‐ 10 N are observed. It is interesting to note that Wang et al.^[^ ^43^ ^]^ are able to overcome the drawback of high power consumption by switching on the magnetic field prior to attachment in order to apply a preload. When adhering to the substrate, the deformation is obtained by switching off the magnetic field. This way, they mimic the elastic energy storage mechanism of the octopus explained in Section 2.2.

### 4.2 Adhesion

**Figure** **10** shows the records' adhesion performance on rough, curved, and deformable surfaces as well as their resistance to a shear load. The table also illustrates which strategies the different records have applied to improve the adhesion performance of the suction cup. To better understand the adhesion strategies, The theory behind the modelling of the attachment force of suction cups will be discussed first^[^ ^50^, ^56^ ^]^ Hwang et al. derived a formula for the theoretical attachment force that accounts for the achieved pressure difference between interior and exterior, the substrate roughness properties and the interfacial interactions between the substrate and the suction cup. The formula is shown below as Equation (1).

(1) $$F=-\Delta P_{0}\cdot \frac{1-\gamma_{\text{flat}}}{\alpha \cdot R_{a}+1}\cdot \frac{\pi \cdot D_{v,\text{in}}^{2}}{4}+\sigma_{\text{rim}}\cdot A^{\prime }$$

In this formula, Δ*P*_0_ is the pressure difference between the interior and the exterior of the suction cup. γ_flat_ is a compensation factor accounting for seal leakage between the suction cup and a completely flat surface. This parameter has a value between 0 and 1. α is the adaptability constant or leakage parameter. Hwang et al^[^ ^50^ ^]^ experimentally determined that this value decreases with a softer infundibulum or the addition of microdenticles. These adaptations allow for better conformation to irregularities or roughnesses in the substrate.^[^ ^57^ ^]^ Continuing on this, *R*_*a*_ is the roughness factor of the substrate. Next, *D*_*v*, in_ is the diameter of the interfacial area. The second part of the formula accounts for the interfacial interactions. σ_rim_ is a parameter describing the interaction forces between the substrate and the suction cup, which accounts for capillary interactions in wet conditions, and Van der Waals forces in dry conditions. Finally, *A*′ is the effective interfacial area. Given all the parameters in this formula, there are several strategies to increase the value of the overall force *F*.

For example, for a suction cup that is softer in the infundibular area (see **Figure** **11**A), it will be easier to conform its shape to the substrate. Hence, this will decrease the leakage compensation factor γ_flat_ and increase the effective interfacial area *A*′. Also, the softness will decrease the leakage parameter α.^[^ ^50^ ^]^ Looking at the formula, these three together will contribute to a higher overall force *F*. The strategy of an infundibular area that is softer than the acetabular region is exploited by several records, as well as in the octopus itself.^[^ ^32^ ^]^

Frey et al.,^[^ ^58^ ^]^ Hwang et al.,^[^ ^50^ ^]^ Lee et al.,^[^ ^49^ ^]^ Song et al.,^[^ ^59^ ^]^ Sandoval et al.,^[^ ^57^ ^]^ Koivikko et al.^[^ ^28^, ^36^ ^]^ and Kortman et al^[^ ^60^ ^]^ applied this principle by attaching a softer silicone membrane (similar to the infundibular surface in the octopus) on the stiffer suction cup body (similar to the acetabular chamber in the octopus), either by using an adhesive,^[^ ^28^, ^36^, ^59^, ^60^ ^]^ a coating,^[^ ^49^, ^50^ ^]^ or by partially curing the membrane before applying it on the suction cup body.^[^ ^57^, ^58^ ^]^ Sareh et al.,^[^ ^61^ ^]^ Wang et al.,^[^ ^43^ ^]^ Shahabi et al.,^[^ ^62^ ^]^ and Wang et al.^[^ ^34^ ^]^ obtained the stiffness difference by molding different types of silicone in batches.

Several records drew inspiration from the surface geometry of the octopus infundibulum, which consists of a network of grooves and radial slits and is covered in a rough microdenticle texture, as explained in Section 2.3. Sandoval et al.^[^ ^57^ ^]^ explain that radial slits improve sealing capabilities by providing more geometric compliance to curved and irregular surfaces (see Figure 11B), In their design, they used radial slits to improve adhesion performance. Sareh et al.,^[^ ^61^ ^]^ Tramacere et al.,^[^ ^14^, ^63^ ^]^ Shahabi et al.,^[^ ^62^ ^]^ and Xi et al.^[^ ^64^ ^]^ also mention experimenting with different geometries for radial grooves and slits.

Next, continuing on the octopus' microdenticle texture, making use of a microstructure in the infundibulum will decrease the leakage parameter α in the formula described above, as explained by Hwang et al^[^ ^50^ ^]^ by making it easier to adapt to rough surfaces (see Figure 11C) and thereby increase the overall force *F*. Also, the improved seal will decrease the leakage compensation factor γ_flat_ and causes an increase in the effective surface area *A*′. This strategy is used by several records.

Li et al.,^[^ ^26^ ^]^ Hwang et al.,^[^ ^50^ ^]^ and Sandoval et al.^[^ ^57^ ^]^ applied this principle through the addition of rough microstructures on the suction cup's infundibular surface. Kim et al.^[^ ^45^ ^]^ achieved this as a beneficial side‐effect of the shrinkage of microchannels in their hydrogel‐based suction cup. The size and geometry of the microchannels create the same effect as a rough microdenticle texture would do.

Another strategy to improve the overall adhesion force is to focus on the interfacial interactions, for which the second part of the formula (σ_rim_ · *A*′) accounts. Making use of an adhesive layer in between the suction cup and the substrate will increase the value of σ_rim_ by increasing Van Der Waals forces, and may also influence the leakage compensation factor γ_flat_ and increase the effective surface area *A*′ (see Figure 11D).

For example, Tsukagoshi et al.^[^ ^47^ ^]^ used a sticky urethane sheet as a suction cup membrane and Okuno et al.^[^ ^65^ ^]^ used hybrid actuation with both direct suction and a membrane for electro‐adhesion. Cao et al.,^[^ ^37^ ^]^ also report that, even without their suction cup being actuated, a passive adhesion force of 0.08 N is achieved due to the stickiness of the membrane that is used.

A final strategy, closely related to having a softer infundibular portion, is actively adapting the suction cup's stiffness to the substrate. Zhuo et al.^[^ ^54^ ^]^ combined membrane suction cups for force generation and heat‐responsive hydrogels for stiffness adaptation. Their suction cup used multiphase hydrogels with programmable stiffness in its membrane that allowed it to adhere to a broad range of surfaces. For example, the suction cup stiffness can be actively decreased while adhering to rough surfaces in order to improve surface compliance (see Figure 11E). However, as the stiffness change of this material is dependent on heating, the responses are relatively slow. Finally, particle jamming^[^ ^25^, ^26^, ^27^, ^29^ ^]^ is also considered as a type of stiffness adaptation in order to adhere to a broader range of objects.

### 4.3 Manufacturing and Materials

Most of the included records using passive or fluidic methods apply a form of silicone molding for manufacturing the suction cup,^[^ ^14^, ^25^, ^26^, ^27^, ^30^, ^31^, ^34^, ^38^, ^49^, ^50^, ^54^, ^57^, ^58^, ^59^, ^60^, ^62^, ^63^, ^64^, ^65^, ^66^, ^67^, ^68^, ^69^, ^70^, ^71^ ^]^ of which some integrate reinforcements to obtain more beneficial material properties,^[^ ^60^, ^67^ ^]^ integrate sensing^[^ ^62^ ^]^ or actuation^[^ ^54^ ^]^ modules, integrate magnetic particles,^[^ ^43^, ^55^ ^]^ or integrate particles for particle jamming cups.^[^ ^25^, ^26^, ^27^ ^]^ The DEA‐actuated suction cups also use a silicone‐molded sheet for the infundibular surface.^[^ ^35^, ^51^, ^53^ ^]^ The deformability of silicone makes it a suitable material for suction cups. Other advantages of this method include the low implementation costs and high repeatability. Moreover, creating the molds is relatively easy as these can often be 3D printed using Fused Deposition Modeling (FDM). However, the process shows long molding times and degassing is required. Three records mention using some form of additive manufacturing (AM) for producing the soft part of their suction cup. Koivikko et al. apply stereolithography (SLA) for manufacturing the suction cup body using a photopolymer resin with a low shore hardness.^[^ ^28^ ^]^ With a resolution of around 10^−2^ mm, SLA printed objects are also significantly smoother than FDM printed objects. Alternatively, Jeong et al. demonstrate AM of suction cups using material jetting.^[^ ^39^ ^]^ This technology is faster and allows for the combination of multiple materials in a single print. This can be exploited to obtain stiffness gradients between the suction cup stalk and membrane.^[^ ^72^ ^]^ Drawbacks are the high material costs and material hysteresis. Finally, Koivikko et al.,^[^ ^36^ ^]^ used Carbon Digital Light Synthesis (DLS) as an AM technique for fabricating the suction cup. In comparison to silicone molding, this process has fewer steps, takes less time and shows better reproducibility.

### 4.4 Tactile Sensing

In **Figure** **12**, it can be observed that only 11 of the suction cup designs included a form of tactile sensing. Aoyagi et al.,^[^ ^68^, ^73^ ^]^ applied a piezoelectric film on the outside of a bellows suction cup to make it work as a force sensor. Doi et al.^[^ ^66^ ^]^ combined a three‐electrode capacitive proximity sensor with a conductive cup which together were able to estimate contact angle and partial contact/push‐in stroke. Huh et al.^[^ ^38^ ^]^ measured the differential pressure between four inner chambers to obtain information about surface curvature, proximity, and texture. In this first iteration of their design, the focus lies on obtaining useful signals when changing surface textures, curvatures and normal vectors. In Lee et al.,^[^ ^69^ ^]^ the same authors continue on these features of their design by developing an autonomous haptic search method. They showcased correcting the lateral positioning error, as well as the rotational alignment error on several unknown objects to be able to successfully pick them up by trial and error. Sareh et al.^[^ ^61^ ^]^ used a fiber optic head to measure proximity and tactile information, for use in motion planning and measuring the firmness state of the anchor. Frey et al.^[^ ^58^ ^]^ used a micro‐LiDAR optical sensor for measuring proximity next to the suction cup to activate the membrane when it approaches an object. Lee et al.^[^ ^49^ ^]^ spray‐coated four strain sensors on the suction cup's outer wall. They used machine learning algorithms to successfully estimate the object's weight and center of gravity from these input channels. Shahabi et al.^[^ ^62^ ^]^ integrated four microfluidic strain sensors, filled with a conductive fluid, into a silicone suction cup. By measuring the resistance through the strain sensors and using this as an input for several machine learning algorithms, they were able to estimate angles, directions, and stiffnesses of substrates. In a follow‐up research with this design, they proved the ability to characterize different materials, in order to adapt the necessary preload according to the stiffness of the substrate.^[^ ^74^ ^]^ The design of Kirsch et al.^[^ ^42^ ^]^ makes use of the self‐sensing properties of SMAs. By measuring the resistance of the SMA element, they deduct the strain, which is used for actuation control of the suction cup. Finally, Horie et al.^[^ ^31^ ^]^ have embedded a temperature sensor in their suction cup during the process of silicon casting. They showed an observable change in temperature when adhering to tissues with a temperature different from the environment. Other than the records by Huh et al.^[^ ^38^ ^]^, Lee et al.,^[^ ^69^ ^]^ Doi et al.,^[^ ^66^ ^]^ Shahabi et al.^[^ ^62^ ^]^ and Lee et al.,^[^ ^49^ ^]^ all records only used a single signal for their sensing principle.

### 4.5 System Integration and Control

In addition to tactile sensing capabilities, autonomous, and intelligent manipulation requires the integration of control as well. Figure 12 shows that this issue is addressed by seven of the records. Kirsch et al.^[^ ^42^ ^]^ used the self‐sensing properties of SMA to keep the spring in a constant position. However, its focus is mainly on saving energy and preventing overheating. Most other records that integrate control, work with threshold values and binary actuation. For example, Aoyagi et al.^[^ ^68^, ^73^ ^]^ used a piezoelectric film on the walls of a bellows suction cup to measure force. When a certain threshold is reached, a solenoid valve is opened. Yue et al.^[^ ^70^ ^]^ used a force sensor in the robot arm mount to activate the membrane when a threshold force is measured. Frey et al.^[^ ^58^ ^]^ activated the suction cup's membrane when the proximity sensor recorded that the sucker was approaching the object. Jeong et al.^[^ ^39^ ^]^ integrated a spring valve in the suction cup that only opened at a certain force value. In fact, Doi et al.^[^ ^66^ ^]^ is the only record that goes beyond the use of thresholds. They implemented sensor‐based control with a three‐electrode capacitive proximity sensor in the suction cup that determined the picking and placing heights and the contact angle. For the intended application of using suction cups for delicate tasks in confined environments, the use in a commercial or industrial setting would be benefited by integrating the suction cups into a larger (soft) system. This was addressed in several of the included records,^[^ ^30^, ^39^, ^58^, ^67^ ^]^ which can be seen in Figure 12. Frey et al.^[^ ^58^ ^]^ implemented an array of their suction cups into a wearable glove, with which they were able to manipulate a wide range of objects underwater. Mazzolai et al.^[^ ^30^ ^]^ put three zones of suction cup designs onto a tendon‐actuated soft robotic arm and demonstrated grasping varied and complex shaped objects in a 70 mm diameter pipe. Hou et al.^[^ ^67^ ^]^ implemented their final suction cup design into an arm skin for a robotic octopus under development. Zhuo et al.^[^ ^54^ ^]^ implemented an array of their organohydrogel‐based suckers onto a pneumatically actuated octopus‐inspired arm and demonstrated its ability to hold a variety of objects. Horie et al.^[^ ^31^ ^]^ placed four of their suction cups on a crawling robot. Tang et al.^[^ ^71^ ^]^ built an amphibious climbing soft robot with two suction cups. Finally, Jeong et al.^[^ ^39^ ^]^ integrated their self‐sealing suction cup modules on a hand exoskeleton to assist and simplify grasping tasks.

Although not all records have physically demonstrated the integration of the suction cup onto a larger arm or system, many designs do have the potential to be integrated. The six metrics described in Section 3 are used to evaluate this potential.

**Figure 13:**
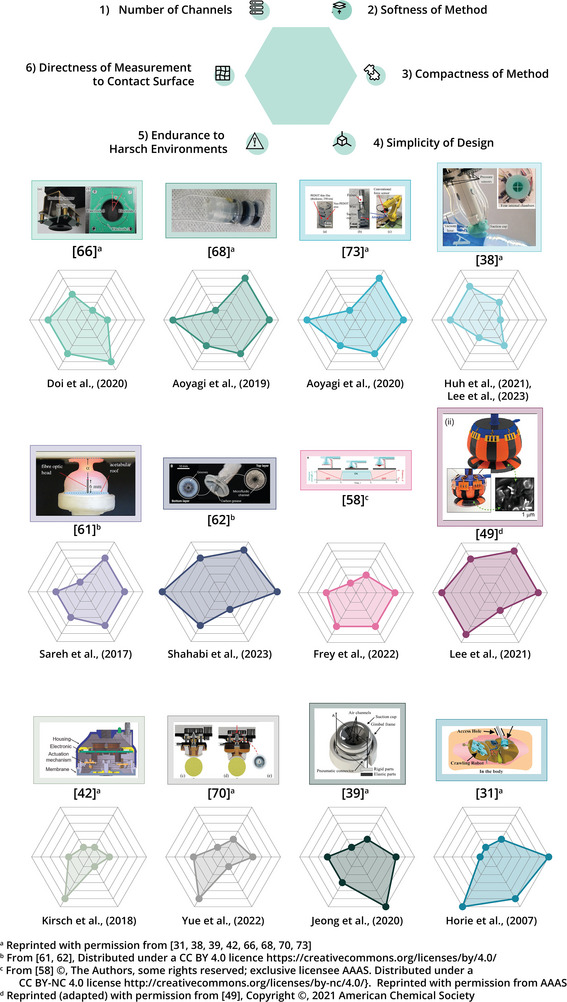
Radar charts representing potential integration of suction cup designs in soft robot arms, elaborated with images from records.^[^ ^31^, ^38^, ^39^, ^42^, ^49^, ^58^, ^61^, ^62^, ^66^, ^68^, ^70^, ^73^ ^]^ A more elaborate figure can be found in Section S4 (Supporting Information).

As sensing and control abilities are considered crucial for arm manipulation, only those records that reported the use of tactile sensing and/or closed‐loop control in their design are included. That is, the records having a green check mark for those metrics in Figure 12. All six metrics have been given a score from one to six, with six indicating the best obtainable score. The scoring procedure can be found back in Section S4 (Supporting Information) attached to this review. The results are visualized in the radar charts in **Figure** **13**. Here, it stands out that Lee et al.,^[^ ^49^ ^]^ and Shahabi et al.,^[^ ^62^ ^]^ score highest overall. This can mostly be traced back to the high degree of integration of their sensing modules in the suction cup's architecture, which makes it score high on the “size”, “softness”, “directness”, and “endurance” metrics. Moreover, they also report the highest number of information channels. Even though Huh et al.^[^ ^38^ ^]^ and Lee et al.^[^ ^69^ ^]^ also use four channels and Doi et al.^[^ ^66^ ^]^ use three, their sensing mechanisms require a large system that is difficult to scale when implementing multiple suckers onto an arm, which lowers their overall score. In general, the softness, size, and directness metrics seem to be relatively poorly addressed. This can be attributed to the fact that most records use rigid sensors that are separated from the body of the suction cup. This results into a low degree of integration of the sensing mechanism in the suction cup's architecture.

## 5 Discussion

In the following subsections, we identify research gaps relating to technology integration, octopus sucker biology, technological limitations, and tests and metrics.

### 5.1 Technology Integration

As explained in Section 3, the performance metrics are divided into three categories. The first category is mainly force‐ actuation‐ and architecture related, the second is about adhesion on a broad range of substrates and environments, and the third is about integration of sensing and control. The octopus sucker outperforms the best performing suction cup in all three categories. Moreover, whereas the octopus sucker performs well on all three metrics, the suction cups presented in most of the records have focused on only one of these categories, with a few exceptions. Huh et al.,^[^ ^38^ ^]^ and Lee et al.^[^ ^69^ ^]^ use the differential pressure between four suction chambers for haptic exploration and report the sensor signals for obtaining a seal on different surfaces. Hereby, two out of the three categories are addressed. However, no attachment forces are measured in this work. Frey et al.,^[^ ^58^ ^]^ use a proximity sensor for activating the suction cup when approaching objects and report high attachment forces of up to 10.5 N. However, the suction cup is not tested on rough surface textures. Shahabi et al.,^[^ ^62^ ^]^ include sensing of substrate direction, angle, stiffness, and inclination. They also report the use of infundibular radial grooves and a stiffness gradient for better adhesion and test their suction cup in both wet and dry environments. However, curved and rough objects are not included in their experiments. The work by Lee et al.,^[^ ^49^ ^]^ is the only work addressing all three objectives. They report using spray‐coated strain sensors on the sucker wall to obtain information about substrate weight and center of gravity. Moreover, their design was tested in both wet and dry environments and attachment to rough surface textures and curved objects was included in their experiments as well. Their reported attachment forces of up to 45 N are the fourth highest out of all included records. It is worth noting that the record with the second highest reported force of 49 N is from the same authors and deploys a nearly identical design that only lacks the microdenticles on the sucker surface.^[^ ^50^ ^]^ A first step toward narrowing down the gap between the octopus sucker and suction cups is to integrate the state‐of‐the‐art technologies for actuation, adhesion (i.e., a combination of the adhesion improving principles illustrated in Figure 11), and sensing in a single suction cup.

Next, the sensorized suction cups should be integrated on soft continuum robot arms. This integration may be challenging for suction cups that rely on external pressure or force sensors^[^ ^38^, ^39^, ^69^, ^70^ ^]^ or make use of rigid sensors that interfere with the suction cup's deformability.^[^ ^31^, ^58^, ^61^, ^66^ ^]^ Finally, the current state‐of‐the‐art is mostly focused on obtaining a sensor signal, but rarely uses the sensed signals for control of the suction cup or robot arm. Records that do integrate closed‐loop control typically only make use of the sensor signal in a binary way by determining a threshold value for actuation of the pneumatic circuit.^[^ ^39^, ^58^, ^70^, ^73^ ^]^ Although this is a meaningful application of the sensed signal, it does not enable precise manipulation strategies or the handling of delicate objects. Although several records mention the use of the sensor signal in control applications as future work, only Doi et al. demonstrate sensor‐based pick and place control with a capacitive proximity sensor in the suction cup^[^ ^66^ ^]^ and Lee et al.^[^ ^69^ ^]^ showcase successfully picking up unknown objects by using an autonomous haptic search method to correct positioning and rotational errors.

### 5.2 Octopus Sucker Biology

Findings from octopus biological studies provide several promising directions towards improving the design of suction cups. For example, mimicking of the octopus infundibular morphology would be a promising direction to realize better resistance to shear forces, as suggested by Tramacere et al.^[^ ^32^ ^]^ They mention that suction cups generally use materials that are quite dissimilar to biological suckers for the infundibular portion. While the commonly used materials are often elastic, the biological tissues show more compliant and visco‐elastic behavior. Soft polyurethanes and hydrogels could be promising materials to explore in this respect.

Considering the octopus' acetabulum, the surface of the sucker interior exhibits high wettability properties. This increases the water cavitation threshold at which bubbles will form and the attachment breaks. The wettability enables the octopus to reach lower negative pressures before the water in the sucker cavitates.^[^ ^9^ ^]^ This property argues for development of suction cups with more wettable internal surfaces to reach higher pressure differences, hence higher attachment forces, which has not received much attention to date.

Third, the hierarchical control architecture explained in Section 2.5 could inspire new planning and control algorithms for soft continuum robot arms with suction cups. The computational costs of these algorithms could be a fraction of the current soft robot control algorithms, yet this topic has remained relatively unexplored. Another property of the octopus sucker that has not received much attention yet, is the energy‐saving mechanism explained in Section 2.2. During the adhesion process, it has been observed that octopuses interlock the acetabular protuberance in the orifice, which allows them to relax their muscles during extended periods of attachment. This interlocking is, for a large part, maintained by the friction caused by hairs on the acetabular protuberance.^[^ ^10^ ^]^ This argues drawing inspiration from this property in the architecture and internal surface properties of artificial suction cups. It could potentially enhance their adhesion performance and energy efficiency.

Finally, gaps exist in our fundamental understanding of the octopus sucker biology. For example, the hairs that were discovered on the acetabular roof^[^ ^75^ ^]^ may have functions beyond adhesion in for example sensing.^[^ ^76^ ^]^ In general, a better understanding of the role that different sensory receptors in the octopus sucker play in the perception of the environment may aid the development of better sensors for suction cups.

### 5.3 Technological Limitations

This subsection will discuss limitations with respect to actuation, sensing, and fabrication technologies that hold back the development of better suction cups.

It should be noted that some of the reviewed actuation technologies are more mature than others, resulting in differences in performance that may not reflect the true potential of the actuation approach well. For example, fluidic actuation methods are widely applied and more mature than methods relying on EHD or IPMCs.^[^ ^40^, ^41^ ^]^ Whereas the latter methods may have focused on demonstrating the viability of the actuation principles for generating a vacuum in suction cups, works on the fluidically actuatated suction cups may have focused on optimizing the design for surface adhesion and attachment force. Another technological limitation with respect to actuation is the difficulty to mimic the principle of muscular hydrostats. It was explained that the octopus sucker is a muscular hydrostat capable of locally and actively adapting its shape and stiffness. Zou et al.,^[^ ^77^ ^]^ mention that achieving this multiple‐mode actuation is almost never obtained in suction cups because these are mostly controlled by a single actuator, such as a pump or voltage source. The remaining material works as a passive limiting structure. Even if an actuation technology allowing for the mimicking of muscular hydrostats becomes available in the future, the control of such actuators would be complex.

The second type of technological limitation relates to the limits of our fabrication technologies. For example, the size of the microdenticles present in octopus suckers reaches down to 2 µm, while the smallest artificial denticle size found in literature was found to be 30 µm.^[^ ^50^ ^]^ Moreover, the octopus sucker seamlessly integrates micro‐scale and macro‐scale structures. Cross‐scale fabrication technologies for mimicking such multi‐scale structures are not yet available. The limitation related to the resolution of fabrication technologies is also clear in the integration of tactile sensing principles. While the octopus sucker exhibits more than 10000 receptors in a single sucker of 3 mm diameter,^[^ ^13^ ^]^ the highest number of sensors found in literature was four.^[^ ^38^, ^49^, ^62^, ^69^ ^]^ This limitation could partially be overcome by transferring the principles of tactile sensing skins to suction cups. Roberts et al.,^[^ ^78^ ^]^ mention that E‐skins are often completely soft and therefore hardly interfere with an impedance of underlying structures and contact mechanics. They could provide information about contact, shape, texture, forces, and deformation. However, the resolution of state‐of‐the‐art tactile skins is still far from the receptor density in an octopus sucker.

### 5.4 Tests and Metrics

Mechanical tests, such as stiffness, roughness, and shear resistance tests, are critical for evaluating the performance of suction cups in robotic manipulators. However, most of the research on suction cups has focused on the first two types of tests, neglecting the importance of shear load resistance. There was one exception that solely focused on shear forces, which was the design that integrated two suction cups in a climbing robot made by Tang et al.^[^ ^71^ ^]^ As their design goal is to create a climbing robot that is able to walk on vertical walls, shear forces are their main focus and normal pull‐off loads are not discussed. Besides this study, three other studies^[^ ^47^, ^57^, ^65^ ^]^ reported measurements of shear load resistance, and of those, only two showed satisfactory results.^[^ ^47^, ^65^ ^]^ Although shear loads may be of lower importance when using a manipulator with multiple cups applying forces from various directions, it remains an essential factor to consider when evaluating the individual suction cups.

This review attempted to report the results from all records in a fair, transparent and unbiased manner. However, it should be noted that differences in test methods and focus may have put certain records in a more positive light than others. For example, while some records measured attachment force by pull‐off tests and force sensors, others added incremental weights to assess this metric and did not test until failure. Moreover, degrees of roughness, curvature, and deformability varied across records.

To enable comparison of future suction cup designs across records without ambiguity, it is recommended to develop standard testing methods to assess the most important metrics. As also proposed by Croll et al.,^[^ ^79^ ^]^ these metrics and their measurements should be independent of the suction cup's size. We propose the following experiments to be conducted for each design. First, a test to measure the attachment force in both the normal and shear direction should be conducted. However, the value of attachment force in the normal direction *F*_*n*_ is heavily affected by the preload force used in the experiment.^[^ ^58^ ^]^ Therefore, a range of values for the preload force *F*_*p*_ should be applied until the highest attachment force in the normal direction *F*_*n*, *optimal*_ has been determined. The same procedure should be applied for determination of attachment force in the shear direction *F*_*s*_. We recommend both of these tests to be conducted in both a dry and wet environment, while using standardized substrates in a range of predetermined roughness values. An example can be found in the work of Zhuo et al.,^[^ ^54^ ^]^ where objects with average roughness of *R*_*a*_ = 0, *R*_*a*_ = 20, *R*_*a*_ = 50 and *R*_*a*_ = 200 µm were used. For size independence, it is recommended to conduct the same experiments on curved substrates with diameters based on the suction cup's diameter. A good guideline for selecting the diameter ratios can be found in the work of Yue et al.,^[^ ^70^ ^]^ where it is observed that the attachment success rate of their design ranges from 0% to 100% when the substrate diameters are chosen in a range between 1/2 to 1/3 times the diameter of the suction cup itself. Next, in order to compare the determined force values across multiple suction cups unambiguously, it is recommended to normalize the attachment forces by dividing their values by the suction cup's weight *m* and interfacial area *A*, which would result in normalized ratios. The first set of ratios would be the strength‐to‐weight (*STW*_*n*_ = *F*_*n*, *optimal*_/*m* and *STW*_*s*_ = *F*_*s*, *optimal*_/*m*) and the second set would be the stress in the normal and shear direction (σ_*n*_ = *F*_*n*, *optimal*_/*A* and σ_*s*_ = *F*_*s*, *optimal*_/*A*). Finally, it is recommended to measure the response time by determining the time needed to switch between the ON and OFF state of the suction cup (*T*_*switch*_). The conduction of all of these described experiments would provide normalized values, independent of geometry and size.

## 6 Conclusion

This review aimed to analyze the state‐of‐the‐art in the development of suction cups. Existing designs were classified in terms of their actuation technology. Metrics for evaluation were obtained from the capabilities of the octopus sucker and the envisioned future application of integration of suckers in soft robot arms. It is observed that the state‐of‐the‐art makes use of five main classes of actuation methods. These are mechanical or passive methods, fluidic methods, electric methods, thermal methods, and magnetic methods. Fluidic and passive actuation methods currently make up the largest part of the state‐of‐the‐art. With future applications in mind, fluidic methods are preferred because of tunable attachment forces and the possibility of controllable detachment.

Suction cups with high attachment forces and the ability to adhere to a broad range of substrates have already been realized. However, due to a lack of integration of sensing and control, most designs are not yet ready for integration in soft robot arms. Here, possibilities have been demonstrated, but the technologies are not mature enough for industrial applications. In addition to providing recommendations for future work, the evaluation metrics and tests presented in this review work provide a useful framework for evaluation of suction cups in upcoming works.

## Conflict of Interest

The authors declare no conflict of interest.

## Supporting information

Supporting Information
