# Supplementary material for: Classification and Evaluation of Octopus‐Inspired Suction Cups for Soft Continuum Robots
Source: Adv Sci (Weinh). 2024 Jun 14;11(30):2400806. doi: 10.1002/advs.202400806 (PMC11321698; doi:10.1002/advs.202400806)
Supplement: Supplementary file 1 — Supporting Information [file ADVS-11-2400806-s001.pdf]

ADVANCED SCIENCE
Open Access

Supporting Information

for *Adv. Sci.*, DOI 10.1002/advs.202400806

Classification and Evaluation of Octopus-Inspired Suction Cups for Soft Continuum Robots

Stein van Veggel, Michaël Wiertlewski, Eugeni L. Doubrovski, Adrie Kooijman, Ebrahim Shahabi, Barbara Mazzolai and Rob B. N. Scharff**

S1: Search Strategy

The goal of the search strategy was to find papers about artificial suction cups in the field of soft robotics. It was intended to only include papers that described an actual design and experiment of such a device. Therefore, records that only described the theory and/or modelling of such a device were not included in the final review set. Moreover, because the goal of the review was to compare the existing designs using straightforward metrics such as adhesion force, response time and substrate properties, it was decided to only include designs of single suction cups. Sucker sheets with miniature suction cups, or robot grippers that happened to include multiple suction cups, were not taken into account as these cannot be evaluated in the same manner.

The procedure to find records about artificial suction cup designs is illustrated in Fig. A. It was decided to use Google Scholar, Scopus and IEEE Explore as search engines because these were shown to provide the most relevant results in this field.

For building the search query, the terms 'artificial', 'suction cup' and 'soft' were used as a starting point. For these terms, synonyms have been used to come up with the search queries. The used synonyms are indicated below.

- Suction cup: sucker, vacuum cup, suction device, vacuum manipulator, suction manipulator
- Artificial: robotic, robot
- Soft: flexible, compliant, elastic, adaptable

This led to the following search query:

("Suction cup" **OR** "Suction device" **OR** sucker **OR** "vacuum cup" **OR** "vacuum manipulator" **OR** "suction manipulator") **AND** (Artificial **OR** robot*) **AND** (Soft **OR** Flexible **OR** compliant **OR** elastic **OR** adaptable)

The search term was adapted to the specific syntaxes of the search engines to match the query as close as possible. This led to 67, 25400 and 184 results in Google Scholar, Scopus and IEEE Explore respectively. Note that the time of conducting this search was October 2022.

After screening the records by title, 17, 13 and 27 were left on Google Scholar, Scopus and IEEE Explore respectively. On Google Scholar, record titles stopped being relevant after scanning through the first 250 results. Therefore, records after that have not been taken into account. Next, filtering out the overlapping records left 47 records in total. Backward Snowballing added 11 more records to this list. Finally, 15 additional records were added from sources such as recommendations by Academia, ResearchGate, other academic websites, and academics that were involved in writing the review. At this point, 73 records remained.

Screening by abstract excluded 19 records and 54 remained. Next, the full text of the papers was scanned. The criteria described above were used to assess the relevance. This excluded 7 more records. In the final review set, 47 records remained. However, two of the records (Shahabi et al., 2023A) (Shahabi et al., 2023B), described two experiments with the exact same design. As this is described most extensively in the 2023A version, this one has put in the follow-up tables as Shahabi (2023). A flowchart of the search strategy can be found in figure A. Also, one record (Lee et al, 2023) was an advancement of another one (Huh et al., 2021) that could not be seen separately. Therefore, these have been put in as a single design consisting of two records.

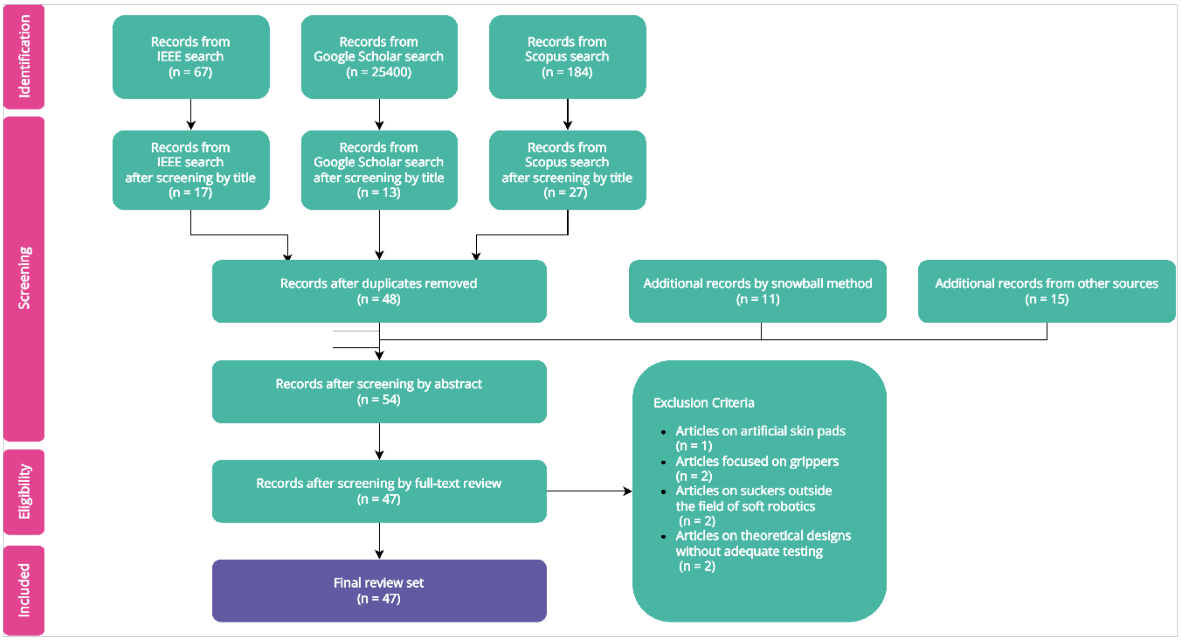

Figure A: Flowchart of the Search Strategy

S2: Classification Procedure

As explained in the main text, the papers were classified according to their actuation method. For this procedure, the actuation for the generation of a pressure decrease was meant. Hence, actuation for the robot arm the sucker was mounted to, or extra actuation technologies for improved adhesion were not involved.

Some records included multiple actuation technologies for generation of pressure decrease. Here, one of the following three procedures was used to classify these.

- If the record included multiple suction cup designs with each a different technology, and the authors clarified that the suction cups were the result of several design iterations or concepts, only the one that was described as best performing, which was often the final one, was included.
- If it was made clear that all suction cups showed comparable performance, and every technology included different advantages and drawbacks, all of them were classified separately. In the result table, this was indicated by stating ‘author et al., (YEARa)’, and ‘author et al., (YEARb)’ etc. for the different designs. Note the difference here compared to suction cups from different records written by the same (first) author and published in the same year, which are differentiated by using capitals letters after the year of publishing (‘author et al., (yearA)’ and ‘author et al., (yearB)’).
- If the actuation technologies were used as hybrid actuation in the same suction cup, both technologies were classified, and the record was included in the table twice. This was indicated with a number in a purple circle next to the reference.

S3: Evaluation Procedure

For obtaining the metrics from the records and filling in the tables, the following procedures were conducted. Regarding the metrics for the table about *General Performance*:

- The *diameter* relates to the outer diameter of the sucker surface that touches the substrate.
- Often, the values for *preload*, *sucker force* and *response time* included multiple values. For example, if multiple experiments or iterations had been performed or if the sucker was tested under different circumstances and/or environments. In these cases, only the highest reported

attachment force, with the corresponding values for preload and response time were included. Also, one record only reported a force that was the result of a climbing robot consisting of two suckers (Tang et al., 2018). The force has been divided by two in this case.
- Although the metric related to being tested in a *dry environment* is relatively straightforward, the check for a *wet environment* required some more explanation. Here, only suction cups that were tested fully immersed in fluid were regarded as complying to this metric. Designs only adhering to a wetted surface or making use of a liquid film did not obtain a check mark here.
- The metric for *no energy use during attachment* was not tested and/or reported in every record. In those cases, the assumed/theoretic energy use of the actuation method during periods attachment was used. For example, designs utilizing fluidic actuation could theoretically turn off their pump after a closed seal with the substrate has been achieved, and designs relying on material deformation by a voltage should theoretically keep applying this voltage to maintain the pressure difference.
- As for the metric *controllable detachment*, the same procedure was used. If nothing was reported, compliance to this metric was assumed if it would be theoretically possible to reverse the adhesion state by e.g. reversing the flow direction of the fluid, or turning off a voltage source.

Regarding the metrics for *Adherence*:

- As for the metric about using a division in *mechanical properties* between a soft sucker surface and a more rigid/elastic sucker chamber, this metric obtained a green check mark only if the sucker chamber could still be considered somewhat elastic. Methods that required fully rigid sucker chambers to e.g. attach electronic components or smart materials (SMA, DEA, IPMC, etc.) were not taken into account because they don't possess the benefits that go along with this metric.
- The *surface microstructure*, *surface geometry* and *stiffness adaptation* metrics are considered straightforward and are assumed not to require any further explanation.
- For the metric about *surface adhesion*, all additional methods which improved adhesion performance, and that could not be classified under the other metrics, were given a green check mark. This included electro-adhesion (Okuno et al., 2019), making use of a sticky material for the suction pad to increase Vander Waals-forces (Tsukagoshi & Osada, 2021), or using a liquid film in between substrate and sucker (Tomokazu et al., 2015).
- For classifying adherence onto *curved*, *rough* or *deformable* surfaces, a loose evaluation procedure was adopted as a great range of substrates was included in the set of experiments. For example, the spectrum of rough surfaces ranged from the surface of an orange to sanding paper and deformable surfaces ranged from thin delicate films to pig hearts. Therefore, broad definitions of 'anything other than fully flat, smooth and rigid' were considered curved, rough and deformable.
- For classifying *shear resistance*, there were only a few records that reported having tested for this. While other records may have been capable of this in theory, making an educated guess was deemed too unreliable. Therefore, a stripe-mark was used if it was not tested for.

Regarding the metrics in the table for *Sensing and Control*:

- For *integration of tactile sensing*, every suction cup using some sort of sensing that provides input about forces and deformations in the suction cup, or about substrate characteristics, was given a green check mark. Additionally, a '~' sign was used to indicate that the suction cup's design would theoretically allow sensing, but this was not specifically applied in the record. For example, the self-sensing properties of DEA's, SMA's and IPMC's would enable deducting the material strain by measuring the resistance and would thus not require any changes to the sucker architecture.

- As for *integration of closed loop control*, actively using some sensor input for actuation of the suction cup was seen as meeting this metric, even if this sensor input was not specifically related to tactile sensing (e.g. input from force sensors on the robot arm or external pressure sensors).
- Although the *metric for number of channels* is considered straightforward as the amount of input data streams can simply be counted, the *softness* and *compactness* of the sensing method can be interpreted in multiple ways. In this case, a cross was filled in if the sensing method required anything rigid or anything that significantly increased the size of the suction cup, compared to using it without sensing the module. To give more nuance in this strict division, these metrics were evaluated on a broader scale later on.
- The metric *integration in larger system* implied using the suction cup in any other method than stand-alone. For example, on a glove, arm, gripper or other kind of robot manipulator.

S4: Creating the Radar Chart for Integration Potential

For the radar chart, it was noticed that evaluating the metrics had a potential to be inconsistent and ambiguous. Therefore, backing arguments have been assigned to every score as seen in the figure on the next page. Following these arguments, a discussion between the authors and an additional comparison between the included records determined the final score. Note that the goal of the figure is to provide the main text of the paper with a qualitative discussion, rather than evaluating the records against a hard-defined criteria.

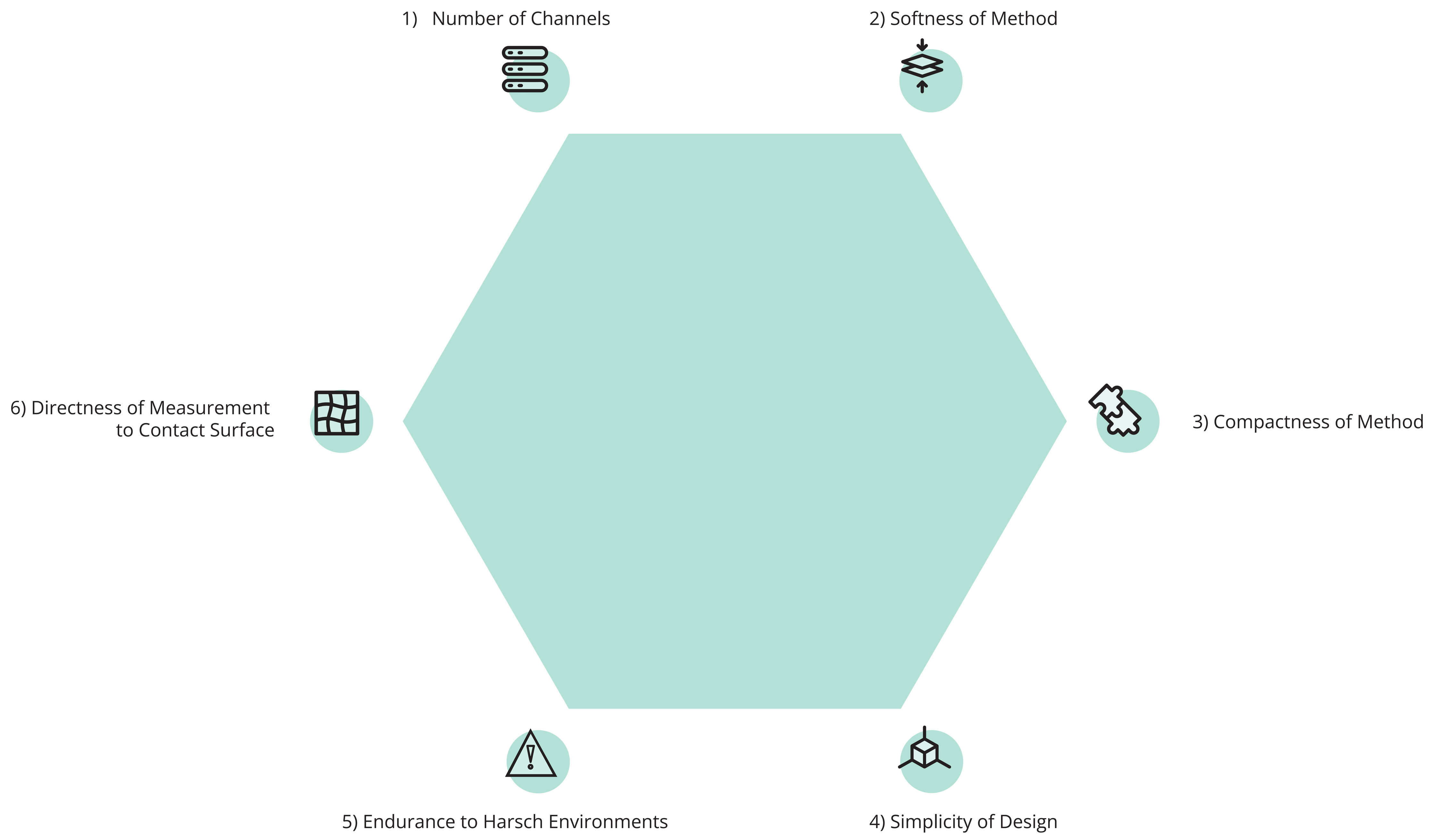

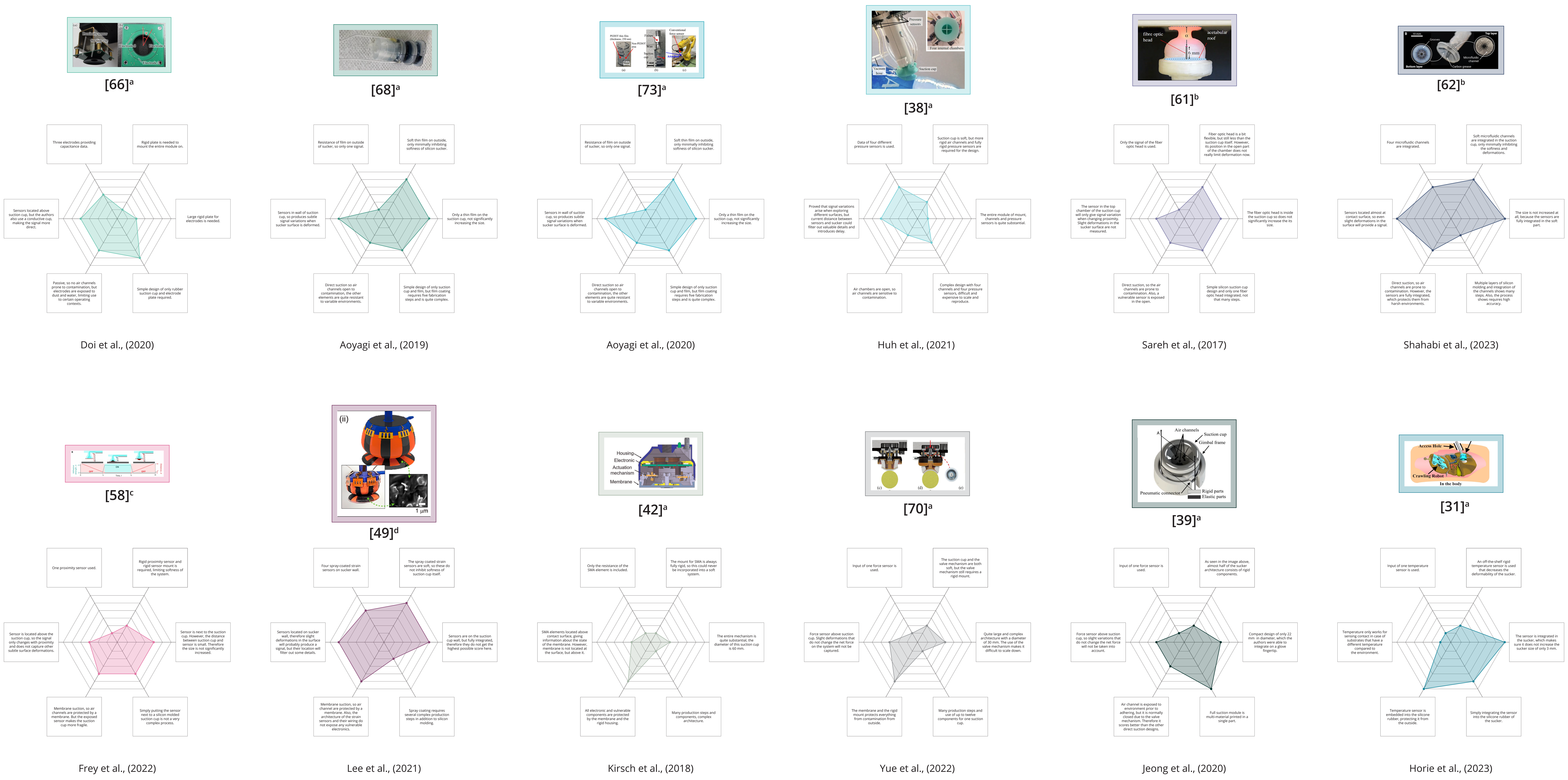

a Reprinted with permission from [31, 38, 39, 42, 66, 68, 70, 73]
b From [61, 62], Distributed under a CC BY 4.0 licence https://creativecommons.org/licenses/by/4.0/
c From [58] ©, The Authors, some rights reserved; exclusive licensee AAAS. Distributed under a CC BY-NC 4.0 license http://creativecommons.org/licenses/by-nc/4.0/}. Reprinted with permission from AAAS
d Reprinted (adapted) with permission from [49], Copyright ©, 2021 American Chemical Society

S5: Raw Data Table

For making the tables for general performance, adherence and sensing & control, the raw data has first been put in the spreadsheet on the next page. This includes additional comments and the search engine for every record.

Referencing						General Properties						Performance						
Paper	In text	Inspiration for Architecture	Actuation	Notes	Scope of paper	Sucker Diameter (mm)	Sucker Height (mm)	Sucker Weight (g)	Testing Environment	Manufacturing	Materials	Generated Negative Pressure (kPa)	Preload for Max Force (N)	Sucker Force (N)	Sucker Force / Area (N/m^2 * 10^2)	Response Time / time before grip is obtained (s)	Energy during suction	Detachment
Doi, S., Koga, H., Seki, T., & Okuno, Y. (2020, 31 May-31 Aug. 2020). Novel Proximity Sensor for Realizing Tactile Sense in Suction Cups. 2020 IEEE International Conference on Robotics and Automation (ICRA),	Doi et al., (2020)	-	External Motor: Passive	They indicated the conductive cup performed better than the non-conductive one, so only the properties of the conductive one have been filled in.	Sensing Principle	35	N.A.	N.A.	Dry	Silicon molding	PFG-35-S/PFG-35-SE from Myotoku (conductive silicon)	N.A.	N.A.	N.A.	N.A.	N.A.	No	Pulling it off
Hou, J., Wright, E., Bonser, R. H. C., & Jeronimidis, G. (2012). Development of Biomimetic Squid-Inspired Suckers. Journal of Bionic Engineering, 9(4), 484-493. https://doi.org/https://doi.org/10.1016/S1672-6529(11)60144-3	Hou et al., (2012)	Octopus	External Motor: Passive	Multiple prototypes were developed, eventually the design that they described as 'final' was performing best, only that one is used in this table.	Sucker shape/geometry	8	N.A.	N.A.	Wet	Type 1: Molding with Reinforcements (knitted nylon sheet reinforced silicon rubber), type 2 and 3: molding	Nylon Sheet, Silicon Ecoflex 0030	N.A.	0,5	2	398	N.A.	No	Applying tensile load until sucker comes off
Mazzolai, B., Mondini, A., Tramacere, F., Riccomi, G., Sadeghi, A., Giordano, G., Del Dottore, E., Scaccia, M., Zampato, M., & Carminati, S. (2019). Octopus-Inspired Soft Arm with Suction Cups for Enhanced Grasping Tasks in Confined Environments. Advanced Intelligent Systems, 1(6), 1900041. https://doi.org/https://doi.org/10.1002/aisy.201900041	Mazzolai et al., (2019c)	Octopus	External motor: Passive	This paper has produced three types of suction cups. Type A, B and C. Performance differed on several aspects but they did not identify one of them as 'best'. Therefore all three have been taken into account. This row describes Type C.	Performance of whole arm where suckers are mounted to	14	N.A.	N.A.	Both	Silicon Molding with reinforcements	Dragon Skin 10 for main body, Ecoflex 0030 for membrane	N.A.	0,5-1	3,1 in air. 1,25 in oil (but unclear whether for entire arm or individual sucker)	201 in air. 81 in oil	N.A.	No	Pulling it off
Sandoval, J. A., Jadhav, S., Quan, H., Deheyn, D. D., & Tolley, M. T. (2019). Reversible adhesion to rough surfaces both in and out of water, inspired by the clingfish suction disc. Bioinspir Biomim, 14(6), 066016. https://doi.org/10.1088/1748-3190/ab47d1	Sandoval et al., (2019)	Clingfish	External Motor: passive	Three components were identified as critical to improve adhesion properties: body geometry, a soft layer in the disc margin, and the presence of slits. Multiple prototypes were developed for this, the one that the authors identified as best was chosen to fill in the properties in this table row (with soft layer, 4 radial slits and soft micropillar structure).	Sucker shape/geometry	25	N.A.	2	Both	Silicon Molding	Silicon Dragon Skin 20 for base, Ecoflex 0030 for sucker sheet	10,1 in air, 14,3 in water	0,5 on smooth. 1,5 on rough	7	143	N.A.	No	Applying shear load or peeling one side
Wang, Y., Sun, G., He, Y., Zhou, K., & Zhu, L. (2022). Octopus-inspired sucker to absorb soft tissues: stiffness gradient and acetabular protuberance improve the adsorption effect. Bioinspir Biomim, 17(3). https://doi.org/10.1088/1748-3190/ac59c6	Wang et al., (2022)	Octopus	External Motor: Passive	Multiple prototypes were developed (material combinations for stiffness gradient varied), the one that the authors identified as best was chosen and that one was filled in this table row.	Sucker shape/geometry and material properties	26	N.A.	N.A.	Both	Molding silicon in batches, in varying hardness	Ecoflex 10-50 (variances)	N.A.	N.A.	9	170	N.A.	No	Pulling it off
Xi, P., Cong, Q., Xu, J., & Qiu, K. (2019). Design, experiment and adsorption mechanism analysis of bionic sucker based on octopus sucker. Proc Inst Mech Eng H, 233(12), 1250-1261. https://doi.org/10.1177/0954411919879358	Xi et al., (2019)	Octopus	External Motor: Passive	Multiple geometries tested, the one that the authors described as best was chosen and only that one is filled in on this table row.	Sucker shape/geometry	35	N.A.	N.A.	Both	Silicon molding	Silicon (specific type not stated)	N.A.	60	9	94	N.A.	No	Pulling it off
Gilday, K., Lilley, J., & Iida, F. (2020, 6-9 July 2020). Suction Cup Based on Particle Jamming and Its Performance Comparison in Various Fruit Handling Tasks. 2020 IEEE/ASME International Conference on Advanced Intelligent Mechatronics (AIM),	Gilday et al., (2020)	-	Fluidic (direct) + Particle Jamming (hybrid)	Hybrid actuation	Actuation mechanism	40	N.A.	N.A.	Dry	Silicon molding of the cup itself, glue to 3D printed base, put coffee grounds in it as jamming particles, separate from vacuum source by fabric filter	Ecoflex 0020 (very compliant)	50	N.A.	19	151	N.A.	No	Release of vacuum
Goshtasbi, A., & Sadeghi, A. (2023). A bioinspired stiffness tunable sucker for passive adaptation and firm attachment to angular substrates [Original Research]. Frontiers in Robotics and AI, 10. https://doi.org/10.3389/frobt.2023.1080015	Goshtasbi et al., (2023)	Octopus	Fluidic (direct) + Particle Jamming (hybrid)	Hybrid actuation	Adaptation to angular surfaces	20	tested with 50-10-20 mm, longest performed best	N.A.	Dry	Silicon molding of suction pad, blade casting thin film of silicon for sucker stalk. Roll thin film around a cylinder, fill cylinder with coffee grains. put a braided filter in the middle of the cylinder to prevent coffee from going in the pump	Different variants, Ecoflex 0010 and Dragonskin 10 for suction pad, Ecoflex 0010 for stalk	60	0.5 N	18,9 (calculated)	602	0.4 s	No	Release of vacuum
Li, J., Song, Z., Ma, C., Sui, T., Yi, P., & Liu, J. (2022). A Bioinspired Adhesive Sucker with Both Suction and Adhesion Mechanisms for Three-Dimensional Surfaces. Journal of Bionic Engineering, 19(6), 1671-1683. https://doi.org/10.1007/s42235-022-00238-5	Li et al., (2022)	Abalone	Fluidic (membrane) + Particle Jamming (Hybrid)	Hybrid actuation	Adhesion microstructures & testing in oil, water and air	19	N.A.	N.A.	Both (+ oil)	Silicon Molding 2 parts and vacuum degassing, assembling them and filling chamber with quartz particles	PDMS for rigid body, PDMS + sylgard 184 for soft membrane	80	3,5	4,3	152	2	No	Release of vacuum
Tomokazu, T., Kikuchi, S., Suzuki, M., & Aoyagi, S. (2015). Vacuum gripper imitated octopus sucker-effect of liquid membrane for absorption. IEEE International Conference on Intelligent Robots and Systems,	Tomokazu et al., (2015)	Octopus	Fluidic (membrane) + Particle Jamming (Hybrid)	Paper describes 21 small suction cups on the sheet of a particle gripper, so it is a bit of an edge case as sucker arrays were originally not taken into account. + Hybrid actuation	Adhesion on many shapes and test with membrane	entire particle jamming sucker 60 mm, individual sucker 6 mm (they used 21)	N.A.	N.A.	Both (+oil), but not immersed, they put some water and oil on the objects	Silicon molding one part, cotton filter pasted over it, fill with glass beads, fix to aluminum jig	KE1308 shin etsu chemical co ltd for elastomer casting, cotton for filter, glass beads as particles	21	20	46	163	N.A.	No	Release of vacuum
Aoyagi, S., Morita, T., Shintani, T., Takise, H., Takahashi, T., & Suzuki, M. (2019, 3-8 Nov. 2019). Formation of PVDF Piezoelectric Film on 3D Bellows Surface of Robotic Suction Cup for Providing Force Sensing Ability - Feasibility Study on Two Methods of Dip-coating and Lamination. 2019 IEEE/RSJ International Conference on Intelligent Robots and Systems (IROS),	Aoyagi et al., (2019)	-	Fluidic (direct)		Sensing	30	N.A.	N.A.	Dry	Silicon molding, and put a PVDF piezoelectric film on the outer mold before closing it (dip coating)	PVDF film, silicon rubber	400	N.A.	N.A.	N.A.	N.A.	No	Pulling it off
Aoyagi, S., Suzuki, M., Morita, T., Takahashi, T., & Takise, H. (2020). Bellows Suction Cup Equipped With Force Sensing Ability by Direct Coating Thin-Film Resistor for Vacuum Type Robotic Hand. IEEE/ASME Transactions on Mechatronics, 25(5), 2501-2512. https://doi.org/10.1109/TMECH.2020.2982240	Aoyagi et al., (2020)	-	Fluidic (direct)		Sensing	10	N.A.	N.A.	Dry	Dip coating the PEDOT film on the silicon suction cup	Commercial silicone rubber suction cup (Schmalz Corp., type: FSG-9-SI-M5) was dip coated with a conductive polymer (PEDOT)	N.A.	N.A.	N.A.	N.A.	N.A.	No	Pulling it off
Gilday, K., Lilley, J., & Iida, F. (2020, 6-9 July 2020). Suction Cup Based on Particle Jamming and Its Performance Comparison in Various Fruit Handling Tasks. 2020 IEEE/ASME International Conference on Advanced Intelligent Mechatronics (AIM),	Gilday et al., (2020)	-	Fluidic (direct) + Particle Jamming (hybrid)	Hybrid actuation	Actuation mechanism	40	N.A.	N.A.	Dry	Silicon molding of the cup itself, glue to 3D printed base, put coffee grounds in it as jamming particles, separate from vacuum source by fabric filter	Ecoflex 0020 (very compliant)	50	N.A.	19	151	N.A.	No	Release of vacuum
Goshtasbi, A., & Sadeghi, A. (2023). A bioinspired stiffness tunable sucker for passive adaptation and firm attachment to angular substrates [Original Research]. Frontiers in Robotics and AI, 10. https://doi.org/10.3389/frobt.2023.1080015	Goshtasbi et al., (2023)	Octopus	Fluidic (direct) + Particle Jamming (hybrid)	Hybrid actuation	Adaptation to angular surfaces	20	tested with 50-10-20 mm, longest performed best	N.A.	Dry	Silicon molding of suction pad, blade casting thin film of silicon for sucker stalk. Roll thin film around a cylinder, fill cylinder with coffee grains. put a braided filter in the middle of the cylinder to prevent coffee from going in the pump	Different variants, Ecoflex 0010 and Dragonskin 10 for suction pad, Ecoflex 0010 for stalk	60	0.5 N	18,9 (calculated)	602	0.4 s	No	Release of vacuum
Huh, T. M., Sanders, K., Danielczuk, M., Li, M., Chen, Y., Goldberg, K., & Stuart, H. S. (2021, 27 Sept.-1 Oct. 2021). A Multi-Chamber Smart Suction Cup for Adaptive Gripping and Haptic Exploration. 2021 IEEE/RSJ International Conference on Intelligent Robots and Systems (IROS),	Huh et al., (2021)	-	Fluidic (direct)	Old version of Lee et al., (2023). Together they have been classified as single design with two iterations.	Sensing	23	N.A.	N.A.	Dry	Silicon Molding	SmoothOn Moldmax 40	50	0,5	N.A.	N.A.	N.A.	No	Release of vacuum
Kortman, V. G., Sakes, A., Endo, G., & Breedveld, P. (2023). A bio-inspired expandable soft suction gripper for minimal invasive surgery—an explorative design study. Bioinspiration & biomimetics, 18(4), 046004. https://doi.org/10.1088/1748-3190/accd35	Kortman et al., (2023)	Octopus	Fluidic (direct)	Uses a membrane, but with a hole in it, so still classified as direct suction instead of indirect.	Suction gripper for surgery	15	N.A.	N.A.	Both	Gravity based dip coating of Nylon Rays and airtight film, then revolving a thin layer of polyethylene foil around, closed footprint is cut open	Nylon and silicon Smooth on 40A	50		6.37 N	39	N.A.	No	Release of Vacuum

	Sensing, Control and System Integration							Adhesion							Force Calculation						
In text	Type of Sensor	Sensor Location	Measured/Sensed Variables	Number of Channels	Application	Control	Arm Integration	Adhesion Improvements	Curved/Flat Substrates	Rough/Smooth Substrates	Deformable/Rigid Substrates	Wet/Dry Adhesion	Shear Stress Resistance		Sucker Diameter (mm)	Sucker Area (mm^2)	Force air	Force water	Force/Area air	Force/Area water	Text
Doi et al., (2020)	Capacitive proximity sensor (self-capacitive digital converter FDC1004) combined with conductive cup	Directly above suction cup	Capacitance	3 (electrodes in circle)	Detecting proximity, contact, contact angle and push-in stroke	Closed Loop	No	None	Flat	Smooth	Rigid	Dry	N.A.		35	962,1	N.A.	N.A.	N.A.	N.A.	N.A.
Hou et al., (2012)	N.A.	Robot mount	Force, Vertical displacement	-	Force experiments	Open Loop	No	None	Flat (metal)	Smooth	Rigid	Wet	N.A.		8	50,3	N.A.	2,0	N.A.	39,8	398
Mazzolai et al., (2019c)	N.A.	N.A.	N.A.	-	-	Open Loop	Yes, in soft robot tentacle actuated by tendons	None	Both (20 mm diameter cylinder)	Smooth	Rigid	Both	N.A.		14	153,9	3,1	1,3	20,1	8,1	201 in air. 81 in oil
Sandoval et al., (2019)	3342, Instron Inc.; capacity 500 N; and 5965, Instron Inc.; capacity 5000 N	Mechanical testing system that cup was mounted to	Force, Displacement	-	Force experiments	Open Loop	No	Softer sheet, Micropillars and radial slits	Both (12.5 mm radius at the smallest)	Both (68 micrometer grain size at the smallest)	Rigid	Both	No		25	490,9	7	N.A.	14,3	N.A.	143
Wang et al., (2022)	2580–2 kN, Instron load cell for force, HT-1891, Dongguan Xintai Instrument Co., Ltd for steady pressure, HM-90G, Nanjing Hong mu Technology Co., Ltd, used for transient pressure	Magnetic cavity	Force, Displacement, Pressure	-	Force/Pressure experiments	Open Loop	No	Radially grooved contact surface of infundibulum, Stiffness gradiënt to make infundibulum softer	Both (cylinder of 42 mm radius at the smallest)	Smooth	Both (pig heart)	Both	N.A.		26	530,9	9	N.A.	17,0	N.A.	170
Xi et al., (2019)	N.A.	No	Force	-	Force performance test	Open Loop	No	Radial grooves in infundibulum, tested different geometries	Flat	Smooth	Rigid	Both	N.A.		35	962,1	9	N.A.	9,4	N.A.	94
Gilday et al., (2020)	SMC sensor, Newton meter	Robot mount	Pressure, Force	-	Force and pressure experiments	Open Loop	No	Particle jamming can be seen as shape adaptation to curved surfaces	Both	Both	Both	Dry	N.A.		40	1256,6	19	N.A.	15,1	N.A.	151
Goshtasbi et al., (2023)	Pressure sensor, Force and position sensor	By pneumatic channels, Robot mount	Pressure, Force, Position/deflection	-	Pressure experiments	Open Loop	No	Particle jamming can be seen as shape adaptation to curved (and in this case angular) surfaces	Flat (but angled)	Both (glass and sandpaper)	Rigid	Dry	N.A.		20	314,2	18,9	N.A.	60,2	N.A.	602
Li et al., (2022)	Hypx 601 force sensor, camera	Robot mount, next to setup	Force, displacement	-	Force experiments	Open Loop	No	2 types of microstructures tested. hexagonal and cylindrical (columnar) + softer membrane	Both	Both	Rigid	Both (+ oil)	N.A.		19	283,5	4,3	N.A.	15,2	N.A.	152
